# KnotAli: informed energy minimization through the use of evolutionary information

**DOI:** 10.1186/s12859-022-04673-3

**Published:** 2022-05-03

**Authors:** Mateo Gray, Sean Chester, Hosna Jabbari

**Affiliations:** 1 Department of Computer Science, University of Victoria, Victoria, Canada; 2 Institute on Aging and Lifelong Health, University of Victoria, Victoria, Canada

**Keywords:** RNA secondary structure, MFE, Pseudoknot, Sequence alignment, Covariation, Thermodynamic energy minimization

## Abstract

**Background:**

Improving the prediction of structures, especially those containing pseudoknots (structures with crossing base pairs) is an ongoing challenge. Homology-based methods utilize structural similarities within a family to predict the structure. However, their prediction is limited to the consensus structure, and by the quality of the alignment. Minimum free energy (MFE) based methods, on the other hand, do not rely on familial information and can predict structures of novel RNA molecules. Their prediction normally suffers from inaccuracies due to their underlying energy parameters.

**Results:**

We present a new method for prediction of RNA pseudoknotted secondary structures that combines the strengths of MFE prediction and alignment-based methods. KnotAli takes a multiple RNA sequence alignment as input and uses covariation and thermodynamic energy minimization to predict possibly pseudoknotted secondary structures for each individual sequence in the alignment. We compared KnotAli’s performance to that of three other alignment-based programs, two that can handle pseudoknotted structures and one control, on a large data set of 3034 RNA sequences with varying lengths and levels of sequence conservation from 10 families with pseudoknotted and pseudoknot-free reference structures. We produced sequence alignments for each family using two well-known sequence aligners (MUSCLE and MAFFT).

**Conclusions:**

We found KnotAli’s performance to be superior in 6 of the 10 families for MUSCLE and 7 of the 10 for MAFFT. While both KnotAli and Cacofold use background noise correction strategies, we found KnotAli’s predictions to be less dependent on the alignment quality. KnotAli can be found online at the Zenodo image: 10.5281/zenodo.5794719

## Introduction

Understanding RNA structure is essential to understanding its function. RNA plays an active role in many processes that occur within the cell, such as in transcription [1], translation [1, 2], splicing [3, 4], catalysis [1, 5] and regulating gene expression [1, 3, 6, 7]. RNA’s function is mainly determined by its structure. As experimental methods are largely expensive for finding these structures, computational methods have become indispensable tools for RNA research.

The majority of computational methods focus on secondary structures—the two dimensional structure of an RNA molecule. Due to similar functions, homologous RNA molecules conserve their common structure. Conservation takes the form of compensatory mutations in response to point mutations that would otherwise cause a change in the structure [8, 9]. Compensatory mutations leave a detectable correlation between positions on a multiple sequence alignment—referred to as *covariation*. Given enough sequences from a related family and an alignment of high structural consistency, comparative sequence analysis (CSA) has been shown to accurately predict secondary structures [10]. Despite the usefulness, circumstances for CSA are limited—homologous sequences and an accurate alignment are not always available especially in cases of novel sequences. A prevalent approach, when such information is not available, is to predict for a single RNA sequence a structure with the minimum free energy (MFE), as structures with minimum free energy are assumed to be the most stable [11]. These programs use a set of empirical parameters to calculate the energy of a structure, where every structural feature has been assigned a specific free energy value. These parameters are not always accurate or known. In addition, these methods assume that an RNA molecule forms a structure in isolation or with minimal interaction with other molecules. These simplifications may result in discrimination between predicted structures and structures found in nature.

Current alignment-based methods couple their covariation with another metric for determining structure and fall into two categories: (1) those that take an unaligned set of sequences and solve the structure and alignment problem concurrently through iterative refinement, and (2) those that take a pre-aligned set of sequences and predict the structure given alignment. Examples of category (1) are algorithms such as locARNA [12–14], FoldAlign [15], MXSCARNA [16], and DAFS [17]. In these algorithms structures of the sequences inform the alignment which, in turn, informs the prediction of the structure. Given the iterative nature of these algorithms they are often more expensive to run than the algorithms in category (2).

Examples of category (2) are algorithms such as RNAalifold [18], Hxmatch [19], Cacofold [20] and Multilign [21]. RNAalifold and Multilign couple their covariation with thermodynamic energy minimization, Hxmatch with maximum weighted matching (MWM), and Cacofold with an RNA-based grammar.

Despite their coupling, these programs still heavily rely on the quality of the alignment to make accurate predictions. In addition, they only predict the consensus structure rather than the structures for all input sequences. Within alignment-based programs, there is an opportunity to address these shortcomings.

In this work we focus on category (2) algorithms and present KnotAli, a novel RNA pseudoknotted secondary structure prediction algorithm which enhances its minimum-free-energy prediction using conserved structural information. Given a sequence alignment of functionally similar RNA molecules, KnotAli finds their individual structures. KnotAli combines two types of information into the prediction. It first uses covariation to find a guide structure and then uses this guide structure to guide the energy minimization step for each sequence that makes up the alignment. We introduce *restricted unpaired bases* and define them as unfavorable bases toward the final structure. We force these bases to be unpaired in our predicted structures.

KnotAli’s prediction accuracy was benchmarked against other existing alignment-based prediction algorithms, two that can handle pseudoknotted structures (Hxmatch [19], and Cacofold [20]), as well as RNAalifold [18] that can only handle pseudoknot-free structures and serves as our control. We note that there are other alignment-based methods that handle pseudoknot-free structures and have similar prediction accuracy based on an independent benchmarking of CompaRNA [22] (see for example, CentroidAlifold [23] and MXSCARNA [16]). In particular, Puton et al. concluded that on average performance of CentroidAlifold and RNAalifold were superior to other comparative-based methods, while the difference on performance of the two was not statistically significant. We chose RNAalifold as the benchmark as Centroidalifold was trained on some of the RNA families included in our dataset (whereas RNAalifold did not need any information in addition to a multiple sequence alignment). We find KnotAli to produce predictions which are more robust to alignment quality deterioration (when compared to Cacofold) and to perform better to a significant degree on the majority of families compared to other algorithms.

## RNA secondary structure

We represent an RNA molecule with its sequence, *S*, and its length *n*. An RNA sequence is made up of four bases: Adenine (A), Cytosine (C), Guanine (G), and Uracil (U). When referring to an alignment of multiple RNA sequences, in addition to the four bases we sometimes observe a “-” (gap) which holds the position of an insertion/deletion (indel) in the alignment. Note that due to indels an alignment might be longer than the RNA sequences—we denote this length as $n_a$.

**Fig. 1:**
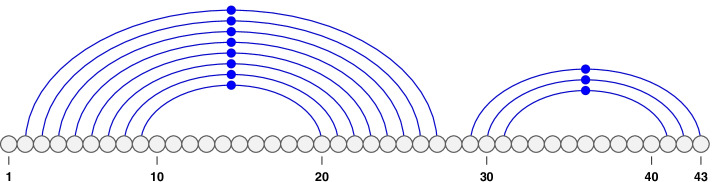
An example of a pseudoknot-free structure. We notice that the stems are non-overlapping. This figure was made using the VARNA software [65]

When an RNA sequence forms a structure, its complementary bases pair together and form hydrogen bonds. ‘A’ pairs with ‘U’ and ‘G’ pairs with either ‘C’ or ‘U’—termed *canonical base pairs*. We refer to bases by their position in *S*. A *base pair* is then defined as the pairing of two bases *i* and *j* where $1 \le i < j \le n$. A base pairing is represented by a “.” (dot). We note that each base can pair with maximum one other base (i.e. no base triplets are allowed). In Fig. 1, we note that the sequence is comprised of 43 bases and each arc signifies a base pairing. We say base pairs $i\cdot j$ and $i'\cdot j'$ are *nested* if $1 \le i< i'< j' < j \le n$, and *disjoint* if $1 \le i< j< i' < j' \le n$. For example, in Fig. 1 base pairs 3.26, 4.25, 5.24, and 9.20 are nested and base pairs 2.27 and 30.42 are disjoint.

**Fig. 2:**
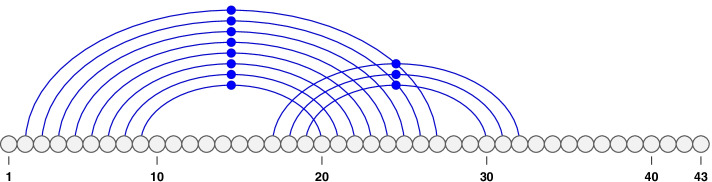
An example of a pseudoknotted structure. Base pairs at 17.32, 18.31 and 19.30 cross the larger stem. This figure was made using the VARNA software[65]

An RNA structure is considered *pseudoknotted* when at least two of its base pairs, $i\cdot j$ and $i'\cdot j'$ cross: $1 \le i< i'< j < j' \le n$, in which case both $i\cdot j$ and $i'\cdot j'$ are considered pseudoknotted base pairs. The example of a pseudoknotted structure shown in Fig. 2 consists of three base pairs at 17.32, 18.31, and 19.30 crossing the larger stem. All base pairs are pseudoknotted within this example. In contrast, structures without crossing base pairs, are called *pseudoknot-free structures*—see Fig. 1. In a pseudoknotted structure, we define a band as the maximal chain of consecutive stacked base pairs with the same crossing patterns. The example pseudoknotted structure in Fig. 2 has two bands: the first is the set of base pairs nested in 2.27 and the second is the set of base pairs nested in 17.32.

**Algorithms.** We start with a high level definition of how the different types of algorithms work and their complexities.

Alignment based algorithms such as RNAalifold [18], Hxmatch [19], and Cacofold [20] measure the interdependence of two columns of an alignment in cubic time. This interdependence measure is then used in one of two ways: 1) merged with the score function for the algorithm or 2) used to select base pairs to inform the later predictions.

Thermodynamics-based algorithms [24–29] find the structure with the minimum free energy for an individual sequence using dynamic programming. Every structure feature is assigned an energy value (some were experimentally determined and others were extrapolated from experiments), and the energy of a structure is calculated as the sum of the energies for each substructure. Consequently, one selects, from the set of all possible structures, the structure whose free energy is minimum. For pseudoknot-free structure prediction, the standard time and space complexity is ${\mathcal{O}}(n^3)$ and ${\mathcal{O}}(n^2)$.

MFE pseudoknotted secondary structure prediction is NP-hard [30, 31] and inapproximable [32]. Polynomial-time algorithms require limiting the class of pseudoknotted structures as time complexity is traded off with generality [11]. The most general thermodynamics-based algorithm is PKnots [26] but it comes with a prohibitively expensive time and space complexity of ${\mathcal{O}}(n^6)$ and ${\mathcal{O}}(n^4)$. While pseudoknot-free MFE-based prediction is sufficient for a subset of RNA, especially smaller molecules, the biological importance of pseudoknots [33, 34] gives cause to developing algorithms that can handle pseudoknotted structures.

It has been shown that the accuracy of MFE RNA secondary structure prediction decreases with sequence length both for pseudoknot-free [35] and pseudoknotted structures [36]. This has motivated research on incorporating available data (e.g. chemical modification/probing, or alignment information) into the prediction algorithms [37, 38].

In this work we aim to address the mentioned shortcomings. Using the coupling of covariation and thermodynamics, KnotAli is capable of finding possibly pseudoknotted structures in ${\mathcal{O}}(Nn^3)$ time and ${\mathcal{O}}(n^2)$ space. KnotAli handles a restricted yet biologically important types of pseudoknots, i.e. kissing hairpins [39] and H-type pseudoknots [40] with arbitrarily nested substructures. More information about KnotAli and class of structures it can handle is provided in “KnotAli algorithm” section.

## Energy model

Many algorithms for the prediction of RNA secondary structures, use a set of parameters to calculate the free energy of the structure. These sets of free energy parameters are called *energy models*. KnotAli uses the energy parameters of HotKnots V2.0 [41], as they are currently the best available energy model for prediction of pseudoknotted structures. The free energy of a loop is dependent on the temperature of the environment as well as the ion concentration. The energy parameters used in this work were derived for a temperature of $37^{\circ }\hbox{C}$ and 1 M salt (NaCl) concentration. These energy parameters are listed in Additional file 1.

## Methods

In this section we provide the description of our algorithm, KnotAli, in “KnotAli algorithm” section. To capture covariation of a given sequence alignment, and detect the intermediary base pairs in KnotAli, we used two metrics: Mutual Information ($\mathtt{MI}$), described in “Mutual information” section, and adjusted mutual information, referred to as $\mathtt{MIp}$ and explained in “Adjusted mutual information” section. As mentioned in “Introduction” section the focus of our manuscript is on algorithms that take a pre-aligned set of sequences and predict the possibly pseudoknotted secondary structure given the alignment. Therefore, in our comparison we used the only two algorithms of this category that can handle pseudoknotted structures, namely Hxmatch and Cacofold. We included RNAalifold as control. We provide a brief description of RNAalifold, Hxmatch and Cacofold, in “RNAalifold, Hxmatch and Cacofold” sections, respectively.

### Mutual information

Mutual Information or $\mathtt{MI}$ is the reduction in uncertainty of one position given another. It can be thought of as a measure of mutual dependence between two columns in an alignment. Measured in bits, the range of $\mathtt{MI}$is between 0 and 2, where 0 suggests no detectable dependency between the two positions and 2 suggests a high dependency. Due to the effect of compensatory mutations, positions with conserved base pairs have a higher dependency on each other than independent positions. $\mathtt{MI}$ is used to find these conserved base pairings.

Our mutual information function is adapted from the MIToolbox [42]. In a standard mutual information calculation, 4 bases and a gap would allow for 25 possible pairs. Only 6 of these pairs form valid base pairs (canonical base pairs). When calculating $\mathtt{MI}$  we ignore non-valid pairs. Let $f_{a,b}(x,y)$ denote the joint frequency of bases *x*, *y* at columns *a*, *b* of the alignment respectively; similarly, let $f_{a}(x)$ denotes the frequency of base *x* at column *a* and $f_{b}(y)$, the frequency of base *y* at column *b*. We define the *mutual information* between column *a* and column *b* of an alignment, denoted *MI*(*a*, *b*), as follows:

1 $$\begin{aligned} MI(a,b) = \sum _{x,y \in \{ A,C,G,U \}} f_{a,b}(x,y) \cdot \log _2\left( \dfrac{f_{a,b}(x,y)}{f_{a}(x) \cdot f_{b}(y)}\right) \end{aligned}$$

### Adjusted mutual information

Adjusted mutual information or $\mathtt{MIp}$ is the reduction of uncertainty of one position given another when taking into account the effect of noise. While $\mathtt{MI}$ works well at finding column interdependence in an alignment, it suffers from noise due to random and phylogenetic sources [43]. The reduction of noise has been shown to improve measures of covariation [44]. Average Product Correction, $\mathtt{APC}$, was previously applied to remove background noise in protein structure prediction [43].

The average product correction for columns *a* and *b* of a given sequence alignment is defined as:

2 $$\begin{aligned} APC(a,b) = \dfrac{MI(a,{\bar{z}}) \cdot MI(b,{\bar{z}})}{MI_{\mathrm {avg}}} \end{aligned}$$

where

3 $$\begin{aligned} MI(a,{\bar{z}}) = \dfrac{1}{n-1}\sum _{z=0}^{n-1} {\left\{ \begin{array}{ll} MI(a,z) &{} \text {where}\,|a-z |> 3 \\ 0 &{} \text {otherwise} \end{array}\right. } \end{aligned}$$

and

4 $$\begin{aligned} MI_{\mathrm {avg}} = \dfrac{2}{n(n-1)} \sum _{w=0}^{n-1}\sum _{z=0}^{n-1} {\left\{ \begin{array}{ll} MI(w,z) &{}\text {where}\, |w-z |> 3 \\ 0 &{} \text {otherwise} \end{array}\right. } \end{aligned}$$

The adjusted mutual information, $\mathtt{MIp}$, is then defined as the difference between $\mathtt{MI}$ and $\mathtt{APC}$ as follows:

5 $$\begin{aligned} MIp(a,b) = MI(a,b) - APC(a,b) \end{aligned}$$

$\mathtt{MIp}$ was found to be more sensitive and selective compared to $\mathtt{MI}$ in protein structure prediction [43].

To determine at what point the $\mathtt{MIp}$ score demonstrates enough interdependence to detect a base pair correctly, we performed a grid search over the threshold range of $[-0.2,1.5]$ with step size of 0.1. Table 1 illustrates the results of the grid search across 21 different possible thresholds on the 10 RNA families as a heatmap. The value at each cell of the heatmap represents the average F-measure for one of the 10 RNA families at a specific threshold. Table 2, similarly represented the average PPV values. We note that the grid search was performed on base pair information obtained using $\mathtt{MIp}$ before the thermodynamic prediction, when we were blind to the final prediction results. We aimed to choose the highest threshold level with acceptable F-measure. This is to avoid detection of incorrect base pairs from the sequence alignment. As noted in Table 1, increasing the threshold results in a general decline in F-measure among all families, with a sharp decline at the threshold value of 0.4. In Table 2, however, we observe that increasing the threshold generally increases the PPV value in all families, with a considerable change after the threshold value of 0.4 (for more information on choice of threshold see Additional file 2). Considering both tables we chose the threshold of 0.4 for KnotAli. All pairings with $\mathtt{MIp}$ $>0.4$ are compiled into a vector. As pairings with repeated positions are possible, i.e. $i\cdot j$ and $i\cdot j'$, the pairings are sorted by score. Base pairs are chosen based on their scores if both bases are available to pair (i.e. they were not paired with another higher scoring base before). These base pairs make up the guide structure for structure prediction as explained in “KnotAli algorithm” section.

**Table 1:**
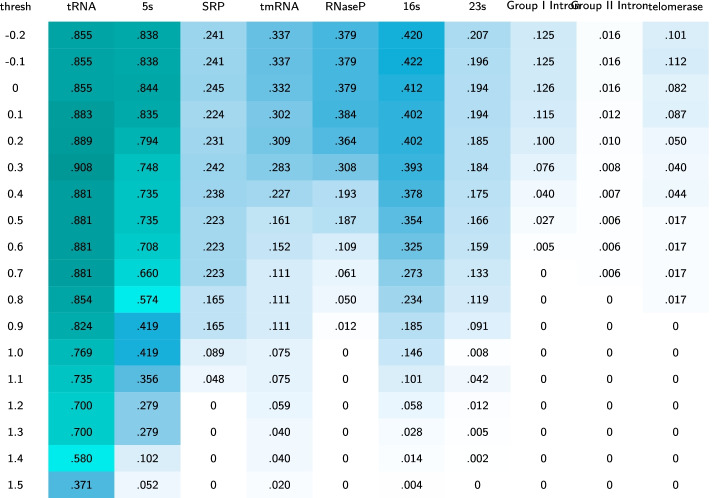
The heatmaps illustrate the results of a grid search across 21 different possible thresholds on the 10 families

The values of the heatmaps represent the mean F-measure for the family at the specific threshold using the MAFFT aligner

**Table 2:**
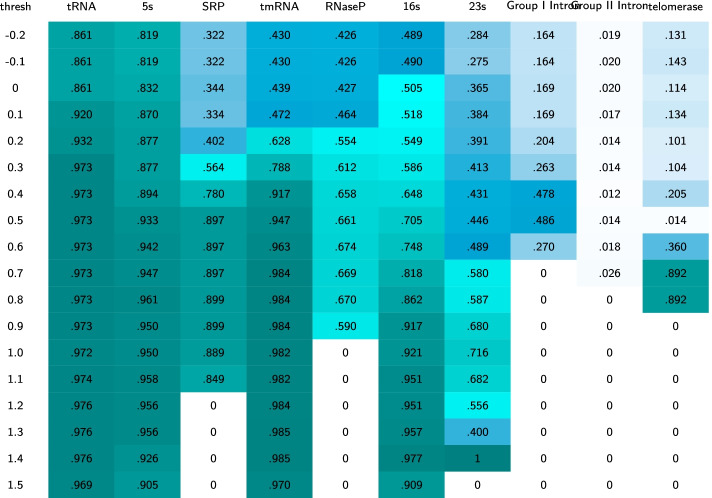
The heatmaps illustrate the results of a grid search across 21 different possible thresholds on the 10 families

The values of the heatmaps represent the mean PPV for the family at the specific threshold using the MAFFT aligner

### KnotAli algorithm

KnotAli’s algorithm incorporates base pair information obtained from conserved structure of homologous RNA sequences into an MFE-based method to predict individual RNA secondary structures for each RNA sequence in the input alignment. In doing so, we bring together three main ideas: 1) selecting a set of intermediary base pairs based on adjusted mutual information, 2) identifying restricted unpaired bases, and 3) relaxed free energy minimization based on a guide structure.

KnotAli uses the average column and alignment mutual information, $MI(a,{\bar{z}}$) and $MI_{\mathrm {avg}}$, respectively (see Equations 3 and 4) to calculate

adjusted mutual information (see Equation 5).

Non-conflicting base pairs with high adjusted mutual information are selected as intermediary base pairs to guide the thermodynamics-based secondary structure prediction step.

In addition, columns whose maximum mutual information is less than the mean mutual information for the alignment are considered as unlikely to pair with any other column, and are marked as *restricted unpaired bases*. The restricted unpaired bases are used to control base pairing within the free energy minimization step.

**Fig. 3:**
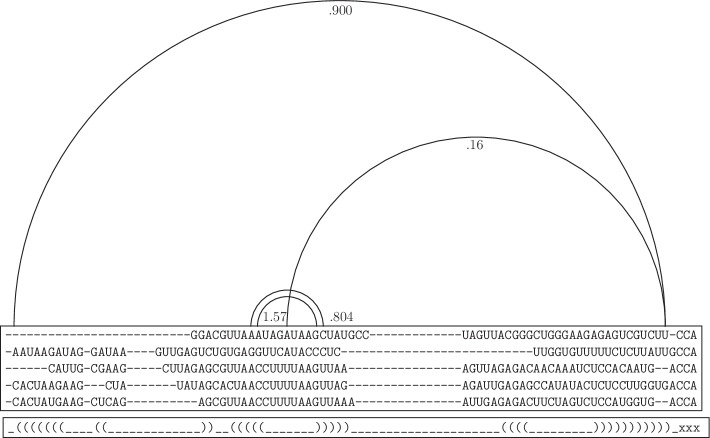
Creating the guide structure in KnotAli. Five sequences are shown from the full alignment of tRNA sequences. Arcs between columns of the alignment represent the $\mathtt{MIp}$ values for the two columns at the two ends of the arcs. Arcs extending the same column represent change of $\mathtt{MIp}$ with a shared column. Here, we only represent a subset of the $\mathtt{MIp}$ values. The bottom line represents the determined guide structure for the alignment. As shown in the guide structure, only non-conflicting base pairs with high adjusted mutual information are selected as the intermediary base pairs, and later included in the guide structure

Combining intermediary base pairs and restricted unpaired bases, we create a *guide structure* for each individual sequence of the alignment to guide its free energy minimization step. Figure 3 shows an example of creating guide structure based on adjusted mutual information. In the guide structure a ‘_’ character is used to signify a base that is free/available to pair with another freely available base, and ‘x’ is used to signify bases that cannot form a base pair. When creating the guide structure for each sequence, we remove bases corresponding to gaps in the sequence and the structure, as well as hairpin loops of size $<3$ that resulted after gap removal [45, 46].

KnotAli follows a relaxed energy minimization step. Relaxed energy minimization was previously used to allow for minor modification of base pairs during a hierarchical folding process, in which an RNA molecule first folds into a simple secondary structure, followed by more complex base pair formation possibly involving base pair competition [25]. Here, we use the relaxed energy minimization approach to allow formation of more stable base pairs for individual sequences of the alignment using the predicted guide structure.

**Fig. 4:**
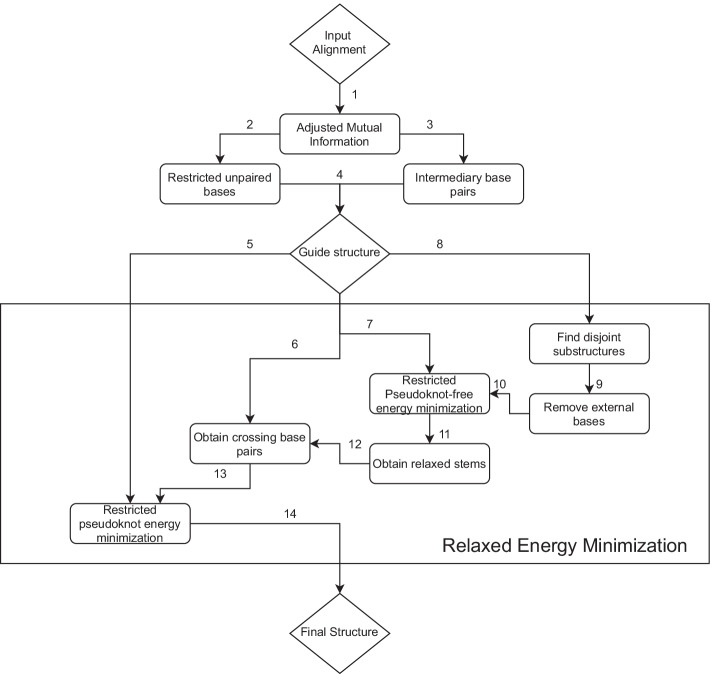
Flowchart of KnotAli. We illustrate the steps taken moving from the input alignment to the guide structure and to the final predicted structure. Note that the *Relaxed Energy Minimization* step is performed once for each sequence of the alignment. There are four methods inside the Relaxed Energy Minimization, that each allow for addition of more stable and possibly competing base pairs with the base pairs of the guide structure. The lowest free energy structure found in the Relaxed Energy Minimization step is output as the predicted structure of a sequence of the alignment. Given an alignment ${\mathcal{A}}$ of *N* sequences, $S_1, S_2, \ldots , S_N$ KnotAli outputs *N* structures $R_1, R_2, \ldots , R_N$

Figure 4 provides a breakdown of each step in KnotAli. KnotAli takes a multiple sequence alignment as input. Adjusted mutual information is calculated based on the sequence alignment and intermediary base pairs as well as restricted unpaired bases are predicted from adjusted mutual information. Combining the intermediary base pairs and restricted unpaired bases for each sequence creates a guide structure for that specific sequence of the alignment. The relaxed energy minimization step consists of 4 different methods, each shown with a different path. These methods are run concurrently. Following *Path 5*, The leftmost method receives the guide structure and finds the possibly pseudoknotted minimum free energy structure given the guide structure (hence called “restricted pseudoknotted energy minimization”). *Path 6* first aims to identify only non-nested (i.e. crossing) base pairs given the guide structure. If found, these crossing base pairs are then provided to the “restricted pseudoknotted energy minimization” to predict the output structure. This is to allow for formation of competing crossing base pairs. Following *Path 7*, first the MFE pseudoknot-free structure given the guide structure is predicted. Then relaxed stable stems (which include stable stems possibly interrupted by small bulges or internal loops) are identified and passed for further modification as in the second method following *Path 12*. This path aims to allow for formation of competing nested base pairs. *Path 8* also aims to allow formation of competing nested base pairs, this time by first opening the loops of the guide structure from outside for each disjoint substructure in the guide structure. Then following the same steps as the third method through *Path 10* (Fig. 3).

**Fig. 5:**
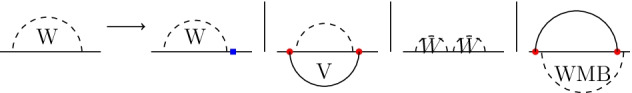
Illustration of the main recursions of restricted pseudoknotted energy minimization step KnotAli. The sequence is represented as a line with bases as index positions; solid arcs indicate base pairs and dashed arcs enclose areas containing unconsumed structure. Red circles represent fixed end points of a region. Blue squares are unpaired indices. We represent the base pairs of the guide structure on top to distinguish then from the predicted base pairs in energy minimization steps of KnotAli shown in the bottom of the line

Restricted minimum free energy structure prediction follows a dynamic programming algorithm depicted in grammar rules represented in Fig. 5. In this grammar rule the RNA sequence is represented as a line with bases as index positions. Solid arcs indicate base pairs, and dashed arcs enclose areas containing unconsumed structure. Red circles represent fixed end points of a structure and blue squares represent unpaired bases. The class of pseudoknotted structures that KnotAli can handle is density-2 structures [47], a subclass of bisecondary structures in which no vertical line drawn at an index position intersects more than two bands. A bisecondary structure is defined as the union of two disjoint pseudoknot-free secondary structures [48]. Restricted energy minimization step in KnotAli’s algorithm receives a pseudoknot-free secondary structure (i.e. the guide structure), and adds a disjoint pseudoknot-free structure to minimize the energy of total structure given the input structure. In *Restricted pseudoknot-free energy minimization* we further restrict addition of base pairs such that the total structure is a pseudoknot-free structure. In *Restricted pseudoknotted energy minimization* step, we make sure that the total structure is a density-2 structure. In our grammar rule representation, as seen in Fig. 5, we present base pairs of the guide structure on top to distinguish them from the predicted base pairs at the restricted energy minimization steps, shown on the bottom of the line.

The energy of the MFE structure between indices *i* and *j* of sequence *S* is calculated by *W*(*i*, *j*) in the dynamic programming algorithm. In case of restricted pseudoknot-free energy minimization, *W*(*i*, *j*) is decomposed into three cases: (1) $W(i,j-1)$ when *j* is unpaired, (2) $V(i,j)+ W(i+1,j-1)$ when *i* and *j* pair together to form a loop (handled by *V*(*i*, *j*)) and the minimum free energy between $i+1$ and $j-1$ is handled by $W(i+1,j-1)$, and (3) $\hbox{min}_{i\le k<j} W(i,k)+ W(k+1,j)$ when there are two disjoint structures available between *i* and *j* that can be handled separately.

In case of restricted pseudoknotted energy minimization step, in addition to the previous three cases *W*(*i*, *j*) has a fourth case which handles pseudoknotted structures (handled by *WMB*(*i*, *j*)), as shown in Fig. 5. *WMB*(*i*, *j*) uses other pseudoknot-specific recurrences (as shown in Additional file 3) to calculate the energy of substructures while separating the guide structure from the predicted base pairs.

The Restricted pseudoknot-free energy minimization follows a dynamic programming algorithm with ${\mathcal{O}}(n^3)$ time and ${\mathcal{O}}(n^2)$ space complexity matching the time and space complexity of the MFE pseudoknot-free prediction algorithms. Since the Restricted pseudoknotted energy minimization step creates a density-2 structure by adding pseudoknot-free base pairs to the guide structure (also pseudoknot-free), its time and space complexity matches the MFE pseudoknot-free prediction as well [47]. KnotAli, therefore, has an ${\mathcal{O}}(N n^3)$ time and ${\mathcal{O}}(n^2)$ space complexity, where *N* is the number of sequences in the input alignment.

### RNAalifold

RNAalifold is a pseudoknot-free consensus structure prediction algorithm which takes a sequence alignment as input. There are two versions of covariation measures that RNAalifold uses within its algorithm.

RNAalifold’s covariation metric, $\gamma$, is defined as

6 $$\begin{aligned} \gamma (a,b) = \gamma '(a,b) + \delta \sum _{S \in A} {\left\{ \begin{array}{ll} 0&{} \texttt{if}\quad S_a.S_b \in \{\texttt{A.U,C.G,G.C,G.U,U.A,U.G}\} \\ 0.25 &{}\texttt{if}\quad S_a\, \texttt{\ and\ }\, S_b\, \texttt{are\ gaps} \\ \texttt{1}&{}\texttt{otherwise} \end{array}\right. } \end{aligned}$$

where *a* and *b* are two columns of the alignment *A* and *S* is a sequence in this alignment. The first covariation score, default setting, uses the Hamming distance as a means of distinguishing possible base pairings. In this case we have

7 $$\begin{aligned} \gamma '(a,b) = \dfrac{1}{2} \sum _{\begin{array}{c} S_1,S_2 \in A \\ S_1 \ne S_2 \end{array}} {\left\{ \begin{array}{ll} h(S_{1_a},S_{2_a}) + h(S_{1_b},S_{2_b})&{} \texttt {if}\quad S_{1_a}.S_{1_b}{} \texttt {\ and\ } S_{2_a}.S_{2_b} \in {\mathbb {B}}\\ \texttt {0}&{} \texttt {otherwise} \end{array}\right. } \end{aligned}$$

Here, ${\mathbb {B}}$ is the set of all possible base pairs, {A.U,C.G,G.C,G.U,U.A,U.G}, and the hamming distance is defined as

8 $$\begin{aligned} h(i,j) = {\left\{ \begin{array}{ll} 1&{} \texttt {if}\quad \texttt {i} \ne \texttt {j} \\ \texttt {0}&{} \texttt {otherwise} \end{array}\right. } \end{aligned}$$

In the second covariation metric referred to as RIBOSUM score, RIBOSUM matrices replace the Hamming distances, as follows

9 $$\begin{aligned} \gamma '(a,b) = \dfrac{1}{2}\sum _{\begin{array}{c} S_1,S_2 \in A \\ S_1 \ne S_2 \end{array}} x\cdot R(S_{1_a}.S_{1_b},S_{2_a}.S_{2_b}) \end{aligned}$$

where *x* is a scaling factor, and *R* is defined as:

10 $$\begin{aligned} R(i\cdot j,i'\cdot j') = \log \left( \dfrac{f(i\cdot j,i'\cdot j')}{f(i,i') \cdot f(j,j')}\right) \end{aligned}$$

$f(i.j,i'\cdot j')$ is the frequency of base pairs $i\cdot j$ and $i'\cdot j'$ being aligned, and $f(i,i')$ (and $f(j,j')$) is the frequency of aligning nucleotides at positions *i* and $i'$ (and *j* and $j'$).

In both cases RNAalifold predicts the MFE structure based on conserved base pairs found using each metric and the energy is adjusted based on a pseudo-energy term that incorporates covariation score.

### Hxmatch

Hxmatch is an alignment-based consensus structure prediction algorithm that can handle pseudoknotted structures. Hxmatch starts by defining a base pair scoring method which combines a *helix score* and a *covariation score*. The helix score, $H^{S}_{i,j}$, considers all possible base pairs for sequence *S* in the alignment and calculates the energy of the largest helix containing the base pair *i*.*j*. The Helix score for two columns *a* and *b* of the alignment *A* is defined as

11 $$\begin{aligned} H^{A}_{a,b} = \frac{1}{N}\sum _{S \in A} H^{S}_{a,b} \end{aligned}$$

The value is multiplied by $-1$ to make it positive and placed in a scoring matrix *H*. The covariation score at positions *a* and *b* is

12 $$\begin{aligned} C_{a,b} =\sum _{i\cdot j,i'\cdot j'} f_{a,b}(i\cdot j)\cdot D_{i\cdot j,i'\cdot j'}\cdot f_{a,b}(i'\cdot j') \end{aligned}$$

where $f_{a,b}(i\cdot j)$ is the frequency of base pair $i\cdot j$ in columns *a* and *b* of the alignment and $D_{i\cdot j,i'\cdot j'}$ is 0 when $i\cdot j = i'\cdot j'$ or either pair is an invalid pairing, equals to 1 when *i* differs from $i'$ or *j* differs from $j'$, and to 2 if both *i* and *j* differ from $i'$ and $j'$, respectively. A penalty is applied to this score based on the number of invalid base pairings at columns *a*, *b*:

13 $$\begin{aligned} B_{a,b} = C_{a,b} - \phi _1\cdot q_{a,b} \end{aligned}$$

where $q_{a,b}$ is the number of invalid base pairings and $\phi _1$ is a scaling factor (default value 0.8) The helix and covariation scores are then combined into a matrix

14 $$\begin{aligned} \pi _{a,b} = H^{A}_{a,b} + \phi _2\cdot B_{a,b} \end{aligned}$$

with $\phi _2$ corresponding to another scaling value (default 60 kcal/mol).

A *maximum weighted matching* (MWM) approach uses the base pair scores found before and builds a set of vertices and edges where vertices are positions from 1 to *n* and the edges are all pairings with a ${\textit{score}}>0$. This step finds the matching which maximizes the sum of edge weights.

### Cacofold

Cacofold is an alignment-based method that can handle pseudoknotted structures. Cacofold uses probabilistic folding methods and positive and negative covariation scores to find a consensus structure. Cacofold is part of the R-scape package [20, 49, 50]. Cacofold uses E-value and covariation power in tandem to distinguish *positive* and *negative* base pairs.

An *E-value* is an expectation value signifying the expected number of false positives [49]. *E* is defined as $E = N\cdot P({\textit{score}}>x)$ where *N* is the number of column pairs and $P({\text {score}}>x)$ is the probability that the column pair would give a covariation score greater than the threshold *x*.

*Covariation power* is an estimate of the expected ability to detect covariations [20, 50]. Covariation power is used to distinguish when a lack of structure is due to low sequence variation rather than low covariation.

A *positive base pair* is a base pair which reports high covariation (a low E-value). In contrast, a *negative base pair* is a base pair which reports low covariation but high covariation power. Negative base pairs are forbidden to appear in the final structure.

Cacofold groups positive base pairs into nested subsets. The first subset is made up of the maximal number of positive pairings such that there are no crossing base pairs or triplets, and succeeding sets are made up of the remaining positive base pairs. The subsets are used as constraints for the secondary structure prediction algorithms. RNA basic grammar [51] is used on the first subset to find the main nested structure. Later subsets use a simplified grammar called G6X, an extension of the G6 model [52, 53], and are used to find additional helices. The structures formed from each subset are combined after filtering out redundancies without covariation support.

## Experiment design

In this section we provide the details of our experiment design.

### Dataset

We tested all algorithms on a large dataset with 3034 (pseudoknotted and pseudoknot-free) RNA sequences. This dataset was compiled from the dataset of [54, 55], by removing duplicate sequences. In addition, we removed all hairpins of size $< 3$ [45, 46]. This step affected the SRP family only. We provide our version of the dataset within the Zenodo image: http://doi.org/10.5281/zenodo.5794719.

The RNA sequences in our database are from ten (pseudoknotted and pseudoknot-free) RNA families with reference structures previously determined through comparative analysis [56]. The pseudoknot-free families are made up of 5s, SRP, Group II Intron, and tRNA while the remaining families contain at least one sequence whose reference structure is pseudoknotted. Sequences vary in length from 28 nucleotides (SRP) to 2968 nucleotides (23s). Sequences in our dataset represent a wide degree of conservation ranging from highly conserved tRNA[57] to less conserved families such as Group I Intron, and Group II Intron [58, 59]. Table 3 summarizes these families.

**Table 3:** List of families with their sequence conservation level, corresponding number of sequences, and range of length

Family	# of sequences	Sequence length	Conservation
5s	1053	103–135	High [66]
16s	22	950–1995	Medium [67, 68]
23s	5	2904–2968	Medium [69]
Group I intron	89	210–736	Low [59]
Group II intron	11	619–780	Low [58]
RNaseP	410	120–486	Medium [70]
SRP	583	28–533	Medium [71]
Telomerase	37	382–559	Low [72]
tmRNA	363	102–437	Medium [73]
tRNA	461	57–93	High [57]

This dataset is comprised of 10 families compiled from RFAM [54–56]

### RNA sequence aligners

To evaluate the structural similarities within differently-sized sequences, the sequences first have to be aligned and gaps placed such that they all have the same length. The strength of a sequence aligner, therefore, plays a fundamental role in the quality of the predicted alignments. In a previous benchmark study [60], 10 different aligners were evaluated. The study sought to score the alignments generated by evaluating the consistency of the secondary structure to the aligned reference sequences. These 10 aligners either predict solely off of sequence similarity or by combining the sequence similarity with structure prediction. Of these 10 aligners, we chose MUSCLE [61] and MAFFT [62], as they solely use sequence similarity.

MUSCLE tends to reduce the number of gaps within the alignment, whereas MAFFT tends to add an increased number of gaps, especially in instances where there is higher variation within the alignment. Both programs only require a FASTA file as input. No additional parameters were used.

### Accuracy measures

We evaluate the performance of algorithms based on three measures: sensitivity (Sen), positive predictive value (PPV), and their harmonic mean (F-measure):

15 $$\begin{aligned} {\textit{Sensitivity}}= & {} \frac{{\textit{TP}}}{{\textit{TP}} +{\textit{FN}}} \end{aligned}$$

16 $$\begin{aligned} {\textit{PPV}}= & {} \frac{{\textit{TP}}}{{\textit{TP}} + {\textit{FP}}} \end{aligned}$$

17 $$\begin{aligned} F_{{\textit{measure}}}= & {} \frac{2\cdot {\textit{PPV}} \cdot {\textit{Sensitivity}}}{{\textit{PPV}}+{\textit{Sensitivity}}} \end{aligned}$$

where the number of *true positives* (TP) is defined as the number of correctly predicted base pairings within the structure; the number of *false positives* (FP), similarly, is the number of predicted base pairs that do not exist in the reference structure; and any base missed in the prediction that corresponds to a pairing in the reference structure is a *false negative* (FN).

Sen, PPV, and F-measure are unitless measures that range between 0 and 1. When the predicted structure is the same as the reference structure, their value is 1. In contrast, when PPV and/or sensitivity is 0, there are no common base pairs between the reference and predicted structure and F-measure is set to 0. High PPV describes an algorithm which predicts a small number of false positives.

In contrast, high sensitivity shows an algorithm’s ability to overall find base pairs from a sequence. Algorithms seek to maximize both. Therefore, combining both sensitivity and PPV helps to better describe the different strengths of algorithms.

### Significance test

We consider the performance of an algorithm to be superior or inferior to another one if the difference in their accuracy is considered significant based on a two-sided permutation test [25, 63]. The two-sided permutation test works as follows. Consider $f_1$ and $f_2$ to be the vectors of F-measures obtained by algorithms $Alg_1$ and $Alg_2$, and $\bar{f_1}$ and $\bar{f_2}$ to be the mean of the F-measures of $Alg_1$ and $Alg_2$, respectively. We term our test statistic $t_s = \bar{f_1} -\bar{f_2}$.

We take samples (with replacement) from vectors $f_1$ and $f_2$ creating a new $f'_1$ and $f'_2$ with the same size as $f_1$ and $f_2$. We recalculate the difference of means between $f'_1$ and $f'_2$ (i.e. $t'_s = \bar{f'_1} -\bar{f'_2}$) and compare it to $t_s$. We repeat these steps 10,000 times. The *p*-value is then the proportion of $t'_s \ge t_s$ out of the 10,000 repeats.

If the calculated *p*-value is less than 0.05, we reject the null hypothesis, concluding that the difference in performance of ${\textit{Alg}}_1$ and ${\textit{Alg}}_2$ is significant. Otherwise we conclude their difference in performance is due to statistical randomness, and thus, not significant. This was accomplished using the ‘perm’ package in R.

### Configuration

The default settings of Hxmatch and Cacofold were used when testing. As mentioned in “RNAalifold” section, there are two options for using RNAalifold, one with Hamming distance as scoring model (default), and one with RIBOSUM scoring model. We assessed performance of RNAalifold with the two scoring models (referred to as RNAalifold for Hamming distance score and Riboalifold for RIBOSUM score) and presented the results in Table 4. In each case we present the results once for MUSCLE and MAFFT as aligners used. Bold font is used to show if there was a significant difference in the results. An asterisk is added when the significance is close enough to warrant distinction but not fully crossing a *p*-value of 0.05. As evident in Table 4 for the majority of families, Riboalifold shows a significant improvement in F-measure over RNAalifold with Hamming distance score. We therefore use RNAalifold with RIBOSUM scoring model when comparing RNAalifold with other algorithms.

**Table 4:** Comparison of RNAalifold with and without the use of RIBOSUM matrices as a covariation measure

Family	MUSCLE						MAFFT					
	RNAalifold			Riboalifold			RNAalifold			Riboalifold		
	Sen	ppv	F	Sen	ppv	F	Sen	ppv	F	Sen	ppv	F
5s	.561	.929	.698	.761	.884	**.817**	.419	.979	.586	.644	.843	**.729**
16s	.323	.818	.462	.505	.748	**.602**	.336	.852	.481	.548	.794	**.647**
23s	.805	.815	**.81**	.798	.767	.782	.807	.806	**.807**	.895	.771	.783
Group I intron	0	0	0	.047	.588	**.087**	0	0	0	.036	.719	**.068**
Group II intron	0	0	0	0	0	0	.105	.992	.189	.106	.773	.186
RNaseP	0	0	0	.235	.602	**.334**	0	0	0	.207	.759	**.322**
SRP	.124	.897	.198	.165	.764	**.241**	.079	.883	.135	.078	.661	.129
Telomerase	.223	.793	.348	.508	.636	**.563**	.225	.869	**.356**	.25	.361	.294
tmRNA	.147	.978	.254	.255	.803	**.386**	.123	.955	.216	.196	.799	**.313**
tRNA	.857	.973	.909	.931	.972	**.949**	.757	.93	.829	.8	.931	.855*

RNAalifold refers to RNAalifold with Hamming distance (default) and Riboalifold is used to denote RNAalifold with RIBOSUM matrices. Results are shown across both MUSCLE and MAFFT. **BOLD** is used to show significant difference in the results. An * is added when the significance is close enough to warrant distinction but not fully crossing a *p*-value of .05

We note the difference in output between our algorithm and the others—namely individual structures versus a consensus structure. For comparison, the consensus structure, from the other three algorithms, is applied to all individual structures. When comparing the results of all algorithms to the reference structure, we did not consider non-canonical base pairs as well as loops of size $<3$ after the removal of gaps.

## Results

Recall that KnotAli receives a multiple sequence alignment as input and predicts individual structures for each of the sequences in the alignment.

To reduce the effect of sequence alignment on performance of KnotAli, we selected two of the best performing sequence aligners, MUSCLE and MAFFT, as explained in “RNA sequence aligners” section. Throughout this work we present KnotAli’s performance with each of the two sequence aligners to provide an unbiased view of its performance.

### $\mathtt{MI}$ versus $\mathtt{MIp}$

**Fig. 6:**
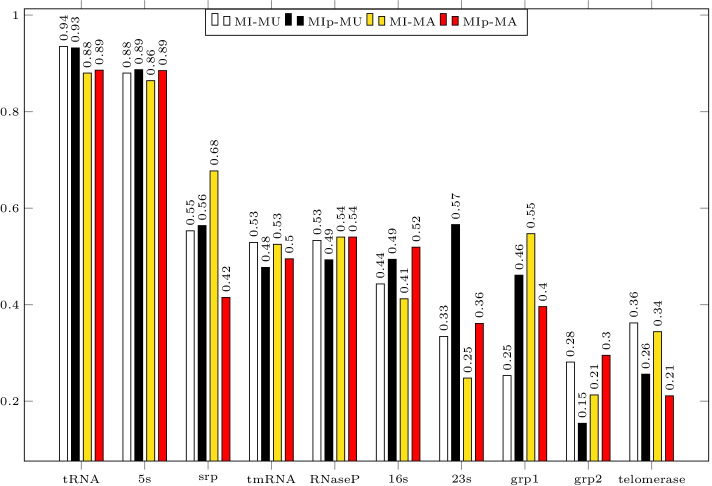
We compare KnotAli’s performance using mutual information, $\mathtt{MI}$, versus its performance using adjusted mutual information, $\mathtt{MIp}$. For each family we present the average F-measure obtained when we used (1) MUSCLE as aligner, and $\mathtt{MI}$ to find conserved base pairs, presented with MI-MU as the leftmost bar in the figure for each family; (2) MUSCLE as aligner, and $\mathtt{MIp}$ to find conserved base pairs, presented with MIp-MU as the second bar from the left in the figure for each family; (3) MAFFT as aligner, and $\mathtt{MI}$ to find conserved base pairs, presented with MI-MA as the third bar from the left in the figure for each family; and (4) MAFFT as aligner, and $\mathtt{MIp}$ to find conserved base pairs, presented with MIp-MA as the fourth bar from the left in the figure for each family

KnotAli’s predictions are guided by the intermediary base pairs—base pair information obtained from covariation in the sequence alignment. To capture covariation we used mutual information as well as adjusted mutual information as it was shown previously that the adjusted mutual information improved accuracy in protein structure prediction [44]. To assess adjusted mutual information ($\mathtt{MIp}$) versus mutual information ($\mathtt{MI}$), we calculated accuracy of the guide structure produced from $\mathtt{MIp}$ and $\mathtt{MI}$ (see Additional file 4). In majority of cases both F-measure and PPV of the guide structures produced using $\mathtt{MIp}$ were significantly higher than those of guide structures produced using $\mathtt{MI}$. Figure 6 presents average F-measure for each family when KnotAli was run in four conditions: (1) MUSCLE as aligner, and $\mathtt{MI}$ to find guide structure, (2) MUSCLE as aligner, and $\mathtt{MIp}$ to find guide structure, (3) MAFFT as aligner, and $\mathtt{MI}$ to find guide structure, and (4) MAFFT as aligner, and $\mathtt{MIp}$ to find guide structure. We compare KnotAli’s results obtained using $\mathtt{MIp}$ to other algorithms.

### Restricted unpaired bases

**Fig. 7:**
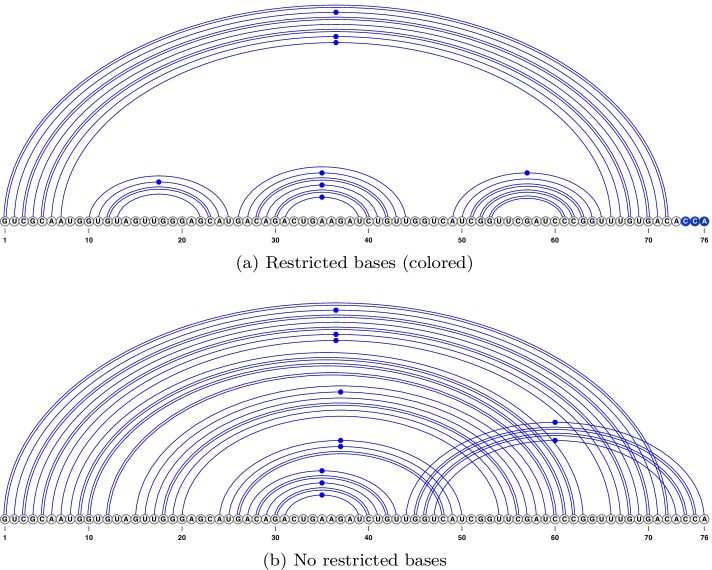
The effect of restricted unpaired bases is shown with an example of an RNA sequence from the tRNA family. If the three bases on the 3’ end of the sequence are free to pair they form pseudoknotted base pairs (**b**) to lower the energy of the structure, therefore causing loss of the cloverleaf shape known within the tRNA family, and deviation from the reference structure. However, once the three bases are identified as restricted unpaired and forced as such (presented as dark filled bases at the 3’ end of part (**a**)) the reference structure is correctly predicted (**a**)

Our second contribution in designing KnotAli is identification of restricted unpaired bases from the sequence alignment. These bases are forced as unpaired in the relaxed energy minimization step of the algorithm. Figure 7 presents an example showcasing secondary structure predicted for an RNA sequence from the tRNA family based on the same intermediary base pairs; in one restricted unpaired bases are identified and enforced (see Fig. 7a in which restricted unpaired bases are identified with a dark fill on the 3’ end of the sequence) and in the second one restricted unpaired bases were not used (see Fig. 7b). When the 3’-end bases are left free to pair, a lower energy structure than the reference structure is predicted as output structure (with free energy of $-12.4$ kcal/mol vs. $-11.88$ kcal/mol of the reference structure). Restricting the 3’-end bases as unpaired (as shown in Fig. 7a) results in prediction of the reference structure for the given sequence. We compared performance of KnotAli with and without restricted unpaired bases, and found a significant difference in favour of using restricted unpaired bases (see Additional file 3). In the rest of this paper KnotAli with restricted unpaired bases is compared with other algorithms.

### Comparison with existing algorithms

**Fig. 8:**
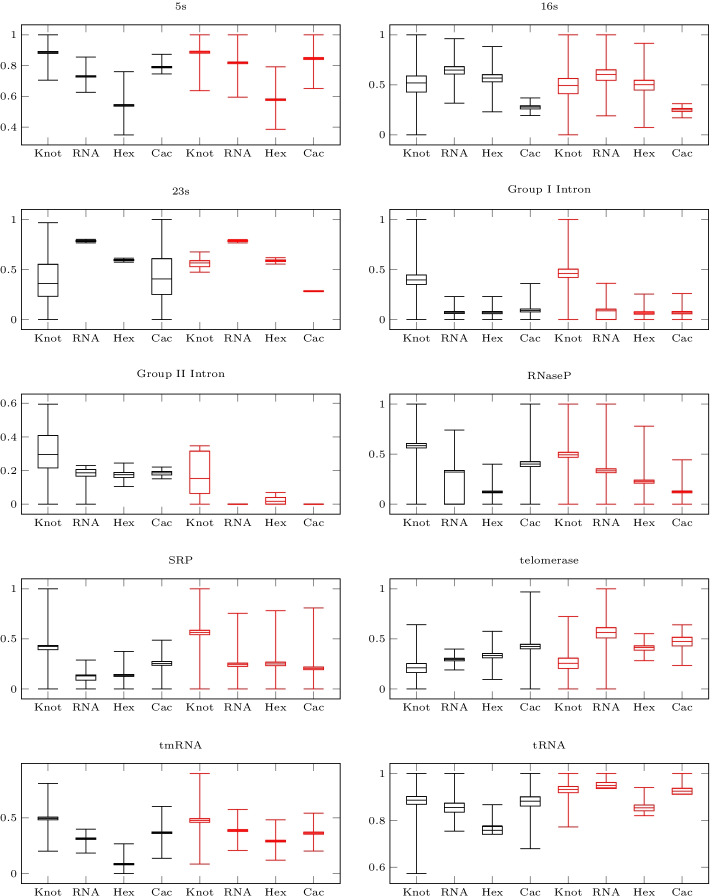
We show the results of the four algorithms in a box and whisker plot (F-measure). Black denotes the results on the MAFFT alignment while red denotes MUSCLE. Algorithms' names are shortened to Knot (KnotAli), RNA (RNAalifold), Hex (Hxmatch) and Cac (Cacofold)

We compared performance of KnotAli (with $\mathtt{MIp}$ for detection of intermediary base pairs, and restricted unpaired bases) with RNAalifold (with RIBOSUM scoring model) which serves as control (RNAalifold takes a multiple sequence alignment as input and predicts a pseudoknot-free consensus structure for the given alignment), as well as Hxmatch and Cacofold both capable of predicting pseudoknotted consensus structures from a multiple sequence alignment. All algorithms were provided with the same sequence alignments for all RNA families in our dataset. Performance of all algorithms are compared once in Table 5a in which sequences were aligned using MUSCLE, and again in Table 5b in which MAFFT was used as the sequence aligner. Bold values in the tables represent significantly superior performers in each family. We find KnotAli’s performance, as measured by F-measure, superior to other algorithms on the majority of the families irrespective of the aligner used (particularly KnotAli performs significantly better than the others on 6 of the 10 families using MUSCLE and on 7 families when using MAFFT as aligner). The results are presented in Fig. 8 as whisker plots, in which black represents the results using MAFFT as aligner and red using MUSCLE.

**Table 5:** Comparison of KnotAli with RNAalifold, Hxmatch, and Cacofold

Family	KnotAli			RNAalifold			Hxmatch			Cacofold		
	Sen	ppv	F	Sen	ppv	F	Sen	ppv	F	Sen	ppv	F
*(a) Input alignment created through MUSCLE*												
5s	.899	.876	**.887**	.761	.884	.817	.424	.917	.579	.835	.859	.846
16s	.494	.501	.494	.506	.748	**.602**	.385	.724	.502	.172	.455	.250
23s	.589	.545	.566	.798	.767	**.782**	.552	.625	.587	.242	.341	.283
Group I intron	.490	.444	**.461**	.047	.588	.087	.034	.529	.064	.039	.300	.068
Group II intron	.177	.139	**.154**	0	0	0	.010	.108	.018	0	0	0
RNaseP	.498	.491	**.493**	.235	.602	.334	.135	.699	.225	.164	.552	.251
SRP	.580	.556	**.564**	.165	.764	.241	.166	.897	.25	.186	.496	.255
Telomerase	.289	.233	.256	.508	.636	**.563**	.292	.711	.413	.380	.480	.423
tmRNA	.491	.468	**.477**	.255	.803	.386	.176	.852	.291	.234	.868	.367
tRNA	.950	.917	.932	.931	.972	.949	.764	.974	.854	.886	.970	.925
*(b) Input alignment created through MAFFT*												
5s	.902	.871	**.885**	.644	.843	.729	.385	.927	.541	.739	.852	.790
16s	.548	.495	.519	.548	.794	**.647**	.440	.802	.567	.198	.467	.277
23s	.329	.468	.361	.795	.771	**.783**	.563	.627	.593	.281	.336	.281
Group I intron	.424	.378	**.396**	.036	.719	.068	.036	.719	.069	.051	.384	.090
Group II intron	.382	.244	**.295**	.106	.773	.186	.103	.580	.175	.105	.920	.187
RNaseP	.592	.583	**.585**	.207	.759	.322	.067	.700	.122	.301	.639	.400
SRP	.420	.416	**.415**	.078	.661	.129	.078	.885	.134	.148	.377	.206
Telomerase	.243	.186	.211	.25	.361	.294	.255	.483	.333	.442	.517	**.475**
tmRNA	.504	.492	**.495**	.196	.799	.313	.047	.435	.084	.255	.650	.362
tRNA	.898	.878	**.886**	.8	.931	.855	.642	.940	.758	.853	.925	.882*

Each column corresponds to algorithm used and each sub-column represents a metric: F-measure, Sensitivity or PPV. **BOLD** represents the significantly highest accuracy compared to others. In the case of two algorithms whose accuracy outperformed the rest while not significantly better than each other, both were represented in bold. An accompanying * is then used to denote a *p*-value close to but not below .05.

Our dataset includes 10 RNA families with varying length, number of sequences in the family, as well as conservation level (see Table 3). In our benchmark, all algorithms performed well on two families, 5s and tRNA both with high level of sequence conservation. Both families included a large number of sequences (1053 sequences in 5s and 461 sequences in tRNA). Reference structures for all sequences in both families are pseudoknot-free. In addition, the length of sequences in both families was relatively short (103–135 in 5s and 57–93 in tRNA). In case of 5s, KnotAli’s performance was significantly better than the rest (with both MUSCLE and MAFFT) while in case of tRNA no algorithm was found to be significantly superior than the others when MUSCLE was used as sequence aligner and KnotAli was found to be the winner when MAFFT was used as aligner (with Cacofold as the runner-up).

Three families in our dataset have low sequence conservation level, namely Group I Intron, Group II Intron and telomerase. These families included a small to moderate number of long RNA sequences (Group I Intron includes 89 sequences of length 210–735, Group II Intron includes 11 sequences of length 619–780, and telomerase includes 37 sequences of length 382–559). Prediction accuracy of all algorithms significantly decreased on these families. In particular, RNAalifold and Cacofold could not find any of the reference structure base pairs (using MUSCLE as sequence aligner) resulting in 0 accuracy in case of Group II Intron, while Hxmatch found few base pairs of the reference structure (accuracy of 0.018). KnotAli’s performance on the same test case was not ideal but was significantly better than the rest, improving Hxmatch’s by over 8-folds. All algorithms performed better on Group II Intron when MAFFT was used as the sequence aligner and KnotAli stayed in the top spot. KnotAli’s performance accuracy was over 5-folds better than the others on Group I Intron with MUSCLE and over 4-folds better than the rest on Group I Intron with MAFFT. On telomerase, however, RNAalifold and Cacofold outperformed KnotAli using MUSCLE and MAFFT respectively.

For the remaining 5 families with moderate sequence conservation level, namely 16s, 23s, RNaseP, SRP, and tmRNA, regardless of the aligner used, RNAalifold performed significantly better on 16s and 23s (respectively with 22 sequences of length 950–1995, and 5 sequences of length 2904–2968) while KnotAli performed significantly better than the rest on RNaseP, SRP and tmRNA (respectively with 410 sequences of length 120–486, 583 sequences of length 28–533, and 363 sequences of length 102–437). We note that all families in this group except SRP had at least one pseudoknotted reference structure in their family. A major difference in performance of RNAalifold and KnotAli on the 16s family is their PPV value (above 0.7 for RNAalifold and around 0.5 for KnotAli). This indicates that KnotAli identifies more base pairs that are not in the reference structure (while addition of these base pairs lowers the free energy value, what KnotAli aims to minimize). In case of 16s, we observe that KnotAli’s sensitivity is similar to that of RNAalifold but its PPV is significantly lower than that of RNAalifold, contributing to its lower F-measure.

### Varying sequence length in alignment

We further assessed the effect of varying sequence lengths in an alignment on performance of all algorithms. We restricted sequence lengths for two families in our dataset with the largest range of lengths. For SRP family with original length range of 28–533 we only compared sequences within 200–350 lengths, and for Group I Intron with original length range of 210–736, we only considered sequences of length between 325 and 450 resulting in 285 and 33 sequences, respectively. The restricted subfamilies were re-aligned using MAFFT and MUSCLE. Results were then compared to the F-measure of structures from the previous prediction (when all sequences were used to create the alignment). As expected all algorithms saw an improvement on their accuracy of prediction on both families when length range of sequences comprising the alignment was tighter (see Table 6). In particular, RNAalifold’s accuracy for SRP increased from .129 to .315 and for Group I Intron from .068 to .205. Hxmatch saw an increase from .134 to .300 for SRP and an increase from .069 to .339 for Group I Intron in F-measure. Similarly Cacofold saw an increase in SRP from .206 to .419 and from .09 to .167 in Group I Intron. The same trend was observed in KnotAli as well: increase in accuracy was observed in SRP from .423 to .581 and from .396 to .546 in Group I Intron. The changes in accuracy for all algorithms were found to be significant when MAFFT was used as the aligner.

**Table 6:** Comparison of KnotAli with RNAalifold, Hxmatch, and Cacofold on two families with large variation in sequence length

family	Pre-shortened				Post-shortened			
	KnotAli	RNAalifold	Hxmatch	Cacofold	KnotAli	RNAalifold	Hxmatch	Cacofold
SRP-MAFFT	.415	.129	.134	.206	.582	.315	.300	.419
Group I intron-MAFFT	.396	.068	.069	.090	.547	.238	.339	.167
SRP-MUSCLE	.564	.241	.25	.255	.491	.198	.232	.200
Group I intron-MUSCLE	.461	.087	.064	.068	.463	.205	.138	.112

Scores are compared between pre-shortened versions and post-shortened versions. Significance is shown between the algorithms on the pre-shortened and post-shortened results. For the significance of the post shortened results to the pre-shortened, see Additional file 3

### Alignment quality effect in KnotAli versus Cacofold

While Cacofold is similar to KnotAli in its use of $\mathtt{APC}$ in background noise correction [20, 64], we saw a distinctly different outcome when comparing Cacofold with KnotAli in their predictions accuracy. To test whether poor quality of the input multiple sequence alignment contributes to the sharp decline in prediction accuracy in Cacofold, we used a known spurious alignment [60] as benchmark to compare KnotAli and Cacofold. We used RF00177 family (bacteria small subunit ribosomal RNA) consisting of 32 sequences with average length of 1476. As control we compared the results with that of 16s family (from our dataset) with 22 sequences of medium sequence conservation, and length range of 950–1995. We present the results in Table 7.

**Table 7:** Comparison of KnotAli to Cacofold using a spurious alignment

Family	KnotAli			Cacofold		
	Sen	ppv	F	Sen	ppv	F
16s	.548	.495	.519	.198	.467	.277
RF00177	.507	.481	.486	.067	.154	.093

Alignment was chosen based on the results of RNAconTest [60]. As control the results are compared to 16s family from our dataset, with similar number of sequences and length range but medium sequence conservation

While KnotAli’s performance on both families is similar (F-measure of .519 for 16s and .486 for RF00177), we observe a sharp decline in accuracy for Cacofold (from .277 in 16s to .093 in RF00177). We therefore, conclude that alignment quality has an observable effect of the performance of both algorithms while KnotAli is less sensitive to it than Cacofold.

### Accuracy measures

We recognize that the reference structures for the sequences within the dataset were determined through comparative sequence analysis [56]. We noted in “Introduction” section that comparative sequence analysis has been shown to accurately predict secondary structures [10]. Structures predicted are not guaranteed to contain all base pairs from the true structure. Within the reference structures of some families provided by comparative sequence analysis, there are large loops indicating a lack of determined structure for the segment. Prediction of stable base pairs (i.e. base pairs with negative free energy value) in some of these segments contributed to decline in PPV value translating to decline in F-measure. We have therefore, compared the accuracy of all algorithms once more when *compatible base pairs* are not considered as false positives in calculation of F-measure.

More specifically, we consider a predicted base pair $i\cdot j$ *inconsistent* if the reference structure includes either $i\cdot k$ or $h\cdot j$ and $h\ne i$ and $k\ne j$ and consider base pairs that are not contradicting as *compatible* if they are not part of the reference structure. Table 8 summarizes the improvements observed in accuracy of all algorithms when compatible base pairs are not penalized. As seen in Table 8, KnotAli outperform other algorithms in 7 families out of 10 for regardless of the aligner used. RNAalifold outperforms KnotAli in 2 families (23s and telomerase) when MUSCLE is used as the aligner. RNAalifold loses to Cacofold on telomerase when MAFFT is used but wins on RNaseP.

**Table 8:** Comparison of accuracy as measured by F-measure for KnotAli with RNAalifold, Hxmatch, and Cacofold when compatible bases are not considered as false positive

Family	MUSCLE				MAFFT			
	KnotAli	RNAalifold	Hxmatch	Cacofold	KnotAli	RNAalifold	Hxmatch	Cacofold
5s	**.920**	.840	.581	.886	**.922**	.765	.550	.832
16s	.560	.635	.527	.267	.593	.677	.588	.297
23s	.639	**.851**	.652	.318	.401	**.851**	.657	.314
Group I intron	**.549**	.087	.065	.079	**.474**	.069	.068	.093
Group II intron	**.205**	0	.018	0	**.431**	.189	.182	.186
RNaseP	**.547**	.341	.225	.255	**.645**	.322	.123	.415
SRP	**.620**	.250	.250	.264	**.463**	.135	.135	.218
Telomerase	.336	**.610**	.430	.479	.278	.325	.361	**.533**
tmRNA	**.556**	.395	.294	.372	**.578**	.319	.087	.373
tRNA	**.961**	**.951**	.854	.926	**.917**	.858	.760	.885

**BOLD** represents the significantly highest accuracy compared to others

Each column represents the aligner used and each subcolumn represents F-measure value for one of the algorithms compared in this work

Comparing the results to Table 5a and b, on families with high level of sequence conservation level (5s and tRNA) KnotAli still outperforms the others on 5s (using both MUSCLE and MAFFT), and tRNA using MAFFT. With the adjusted F-measure, its accuracy significantly improves over the rest of the algorithms on tRNA when using MUSCLE as well. However, improvement in accuracy of Hxmatch is negligible. RNAalifold’s improvement in accuracy is minimal using MAFFT.

For the three families with low sequence conservation level (Group I Intron, Group II Intron and telomerase), KnotAli performs significantly better than the others on Group I Intron and Group II Intron families regardless of the aligner used. While there is a significant improvement in accuracy of KnotAli on telomerase family compared to its previous F-measure, it is not yet able to beat RNAalifold or Cacofold.

In the remaining five families with medium sequence conservation (16s, 23s, RNaseP, SRP, and tmRNA), KnotAli outperforms the rest on RNaseP, SRP  and tmRNA regardless of the aligner used. It is now on par with RNAalifold on 16s family (the previous winner) but still underperforms on 23s when compared to RNAalifold.

## Conclusion

In this work we present KnotAli, a novel algorithm that given a multiple sequence alignment as input predicts the possibly pseudoknotted secondary structure of each RNA sequence within the alignment. KnotAli first identifies a set of intermediary base pairs utilizing a noise adjusted mutual information metric ($\mathtt{MIp}$). Using average mutual information in each column of the alignment, it identifies restricted unpaired bases (the ones that are enforced as unpaired in the guide structure). By combining intermediary base pairs and restricted unpaired bases, it generates a guide secondary structure for each RNA sequence to guide the relaxed free energy minimization step and predicts the individual RNA secondary structure (with possibly pseudoknotted base pairs). We evaluated KnotAli’s performance against a control (RNAalifold) and two competing algorithms (Hxmatch and Cacofold). All algorithms predict their result given a multiple sequence alignment as input. KnotAli, Hxmatch and Cacofold are capable of predicting pseudoknotted secondary structure. While RNAalifold is restricted to pseudoknot-free secondary structures. We benchmarked all algorithms on a large dataset of sequences from 10 families with varying number of sequences, length ranges and levels of sequence conservation using alignments created using MUSCLE and MAFFT. We found KnotAli’s performance to be superior in the majority of the cases. As expected, since all methods compared in this work rely on a multiple sequence alignment provided as input to detect conserved structures, they performed well on two families in our dataset with high conservation level (namely 5s and tRNA), and the accuracy of all methods decreased with a decrease in sequence conservation level. While RNAalifold is not capable of handling pseudoknotted secondary structures, its accuracy was superior to other methods regardless of the aligner used on two families with pseudoknotted reference structures (16s and 23s).

We further compared KnotAli’s performance to Cacofold (that similar to KnotAli utilizes background noise correction strategies and predicts possibly pseudoknotted structures), on a family of sequences with known spurious alignment (RF00177). We found KnotAli to be more resilient to changes in alignment quality compared to Cacofold. While both Cacofold and KnotAli use $\mathtt{APC}$ as a form of background correction, Cacofold uses a G-test covariation measure rather than mutual information (as done in KnotAli). In addition, Cacofold utilizes positive and negative base pairs whereas KnotAli uses intermediary base pairs as well as restricted unpaired bases in its guide structure.

To adjust for inaccuracy in comparative analysis-based reference structures (such as the ones used in this work) caused by large unstructured segments in these structures, we further analyzed performance of all algorithms using adjusted F-measure in which compatible base pairs (those that do not contradict the reference structure) are not penalized as false positive. We observed that KnotAli’s performance further improved compared to other algorithms (only performing worse on the 23s and telomerase families).

Overall, we find KnotAli to provide an improvement over existing methods for prediction of possible pseudoknotted structures from families of functionally related RNAs. We showed that KnotAli performs better that the compared methods in majority of RNA families in our dataset, and is less sensitive to quality of multiple sequence alignment when compared to Cacofold. There is, however, room for improvement. We showed the positive effect of using a better scoring model in the case of RNAalifold (see “Configuration” section), and we plan to improve KnotAli’s scoring model, perhaps by implementing a pseudo-energy term to incentivize retention of intermediate base pairs. Another possible direction is to explore other metrics to detect conservation and covariation in base pairs. These we believe will have significant effect on improving secondary structure prediction for possibly pseudoknotted structures.

## Supplementary Information

**Additional file 1**. Recurrences. We provide the recurrences which make up the thermodynamic MFE prediction within KnotAli.

**Additional file 2**. Energy Table. The table shows the free energy parameters which determine the free energy of the structures.

**Additional file 3**. P-tables. We provide all non-included tables from our paper. Section 1 gives the MI vs MIp comparison table and the associated p-values. Section 2 gives the Restricted vs Non-restricted table and its associated p-values. Section 3 gives the non-included heatmaps for MUSCLE. Section 4 gives the p-values for the comparison with existing algorithms tables included in the text. Section 5 gives the p-values for varying sequence length table. Section 6 gives the p-values for when compatible bases are not considered false positives. Section compares the results between the other algorithms and against themselves.

**Additional file 4**. Cross-validation. We provide our validation results for the heatmap-based threshold pick. Validation results were done through a 70-30 split over 1000 iterations where the threshold was picked based on the 70% training set and assessed on the 30% test set.

## Data Availability

The source code and datasets generated and/or analysed during the current study are available at the Zenodo image: 10.5281/zenodo.5794719.

## References

[1] Cruz JA, Westhof E (2009). The dynamic landscapes of RNA architecture. Cell.

[2] Kozak M (2005). Regulation of translation via mRNA structure in prokaryotes and eukaryotes. Gene.

[3] Mortimer SA, Kidwell MA, Doudna JA (2014). Insights into RNA structure and function from genome-wide studies. Nat Rev Genet.

[4] Warf MB, Berglund JA (2010). Role of RNA structure in regulating pre-mRNA splicing. Trends Biochem Sci.

[5] Wilson TJ, Lilley DMJ (2015). RNA catalysis-is that it?. RNA.

[6] Holt CE, Bullock SL (2013). Subcellular mRNA localization in animal cells and why it matters. Science.

[7] Martin KC, Ephrussi A (2009). mRNA localization: gene expression in the spatial dimension. Cell.

[8] Kirby DA, Muse SV, Stephan W (1995). Maintenance of pre-mRNA secondary structure by epistatic selection. Proc Natl Acad Sci USA.

[9] Wilke CO, Lenski RE, Adami C (2003). Compensatory mutations cause excess of antagonistic epistasis in RNA secondary structure folding. BMC Evol Biol.

[10] Gutell RR, Lee JC, Cannone JJ (2002). The accuracy of ribosomal RNA comparative structure models. Curr Opin Struct Biol.

[11] Mathews DH, Turner DH (2006). Prediction of RNA secondary structure by free energy minimization. Curr Opin Struct Biol.

[12] Will S, Joshi T, Hofacker IL, Stadler PF, Backofen R (2012). LocARNA-P: Accurate boundary prediction and improved detection of structural RNAs. RNA.

[13] Will S, Reiche K, Hofacker IL, Stadler PF, Backofen R (2007). Inferring non-coding RNA families and classes by means of genome-scale structure-based clustering. PLOS Comput Biol.

[14] Raden M, Ali SM, Alkhnbashi OS, Busch A, Costa F, Davis JA, Eggenhofer F, Gelhausen R, Georg J, Heyne S, Hiller M, Kundu K, Kleinkauf R, Lott SC, Mohamed MM, Mattheis A, Miladi M, Richter AS, Will S, Wolff J, Wright PR, Backofen R (2018). Freiburg RNA tools: a central online resource for RNA-focused research and teaching. Nucleic Acids Res.

[15] Sundfield D, Havgaard JH, de Melo ACMA, Gorodkin J (2016). Foldalign 2.5: multithreaded implementation for pairwise structural RNA alignment. Bioinformatics.

[16] Tabei Y, Kiryu H, kin T, Asai K (2008). A fast structural multiple alignment method for long RNA sequences. BMC Bioinform.

[17] Sato K, Kato Y, Akutsu T, Asai K, Sakakibara Y (2012). DAFS: simultaneous aligning and folding of RNA sequences via dual decomposition. Bioinformatics.

[18] Bernhart SH, Hofacker IL, Will S, Gruber AR, Stadler PF (2008). RNAalifold: improved consensus structure prediction for RNA alignments. BMC Bioinform.

[19] Witwer C, Hofacker IL, Stadler PF (2004). Prediction of consensus RNA secondary structures including pseudoknots. IEEE/ACM Trans Comput Biol Bioinf.

[20] Rivas E (2020). RNA structure prediction using positive and negative evolutionary information. PLOS Comput Biol.

[21] Xu Z, Matthews DH (2011). Multilign: an algorithm to predict secondary structures conserved in multiple RNA sequences. Bioinformatics.

[22] Puton T, Kozlowski LP, Rother KM, Bujnicki JM (2013). CompaRNA: a server for continuous benchmarking of automated methods for RNA secondary structure prediction. Nucleic Acids Res.

[23] Hamada M, Sato K, Asai K (2011). Improving the accuracy of predicting secondary structure for aligned RNA sequences. Nucleic Acids Res.

[24] Jabbari H, Wark I, Montemagno C, Will S (2018). Knotty: efficient and accurate prediction of complex RNA pseudoknot structures. Bioinformatics.

[25] Jabbari H, Condon A (2014). A fast and robust iterative algorithm for prediction of RNA pseudoknotted secondary structures. BMC Bioinform.

[26] Rivas E, Eddy SR (1999). A dynamic programming algorithm for RNA structure prediction including pseudoknots. J Mol Biol.

[27] Gruber A, Lorenz R, Bernhart SH, Neuböck R, Hofacker IL (2008). The Vienna RNA websuite. Nucleic Acids Res.

[28] Andronescu M. Algorithms for predicting the secondary structure of pairs and combinatorial sets of nucleic acid strands. University of British Columbia 2003; 10.14288/1.0051269.

[29] Reuter J, Matthews DH (2010). RNAstructure: software for RNA secondary structure prediction and analysis. BMC Bioinform.

[30] Akutsu T (2000). Dynamic programming algorithms for RNA secondary structure prediction with pseudoknots. Discret Appl Math.

[31] Lyngsø RB, Pedersen CN (2000). RNA pseudoknot prediction in energy-based models. J Comput Biol.

[32] Sheikh S, Backofen R, Ponty Y. Impact of the energy model on the complexity of RNA folding with pseudoknots. In: Combinatorial Pattern Matching, pp. 321–333. Springer, Berlin, 2012. 10.1007/978-3-642-31265-6_26.

[33] Uroda T, Anastasakou E, Rossi A, Inga A, Chillón I, Marcia M (2019). Conserved pseudoknots in lncRNA MEG3 are essential for stimulation of the p53 pathway. Mol Cell.

[34] Staple DW, Butcher SE (2005). Pseudoknots: RNA structures with diverse functions. PLOS Biol.

[35] Backofen R, Tsur D, Zakov S, Ziv-Ukelson M (2011). Sparse RNA folding: time and space efficient algorithms. J Discrete Algorithms.

[36] Jabbari H, Wark I, Montemagno C (2018). RNA secondary structure prediction with pseudoknots: contribution of algorithm versus energy model. PLOS ONE.

[37] Hajden C, Bellaousov S, Huggins W, Leonard CW, Mathews DH, Weeks KM (2013). Accurate shape-directed RNA secondary structure modeling, including pseudoknots. Proc Natl Acad Sci USA.

[38] Matthews DH, Disney MD, Childs JL, Schroeder SJ, Zuker M, Turner DH (2004). Incorporating chemical modification constraints into a dynamic programming algorithm for prediction of RNA secondary structure. PNAS.

[39] Melchers WJ, Hoenderop JG, Slot HJB, Pleij CW, Pilipenko EV, Agol VI, Galama JM (1997). Kissing of the two predominant hairpin loops in the coxsackie B virus 3’ untranslated region is the essential structural feature of the origin of replication required for negative-strand RNA synthesis. J Virol.

[40] Alam SL, Atkins JF, Gesteland RF (1999). Programmed ribosomal frameshifting: much ado about knotting!. PNAS.

[41] Andronescu MS, Pop C, Condon AE (2010). Improved free energy parameters for RNA pseudoknotted secondary structure prediction. RNA.

[42] Pocock A, Brown G, Zhao M, Lujan M (2012). Conditional likelihood maximisation: a unifying framework for information theoretic feature selection. J Mach Learn Res.

[43] Dunn SD, Wahl LM, Gloor GB (2008). Mutual information without the influence of phylogeny or entropy dramatically improves residue contact prediction. BMC Bioinform.

[44] Lindgreen S, Gardner PP, Krogh A (2006). Measuring covariation in RNA alignments: physical realism improves information measures. BMC Bioinform.

[45] Danaee P, Rouches M, Wiley M, Deng D, Huang L, Hendrix D (2018). bpRNA: large-scale automated annotation and analysis of RNA secondary structure. Nucleic Acids Res.

[46] Groebe DR, Uhlenbeck OC (1988). Characterization of RNA hairpin loop stability. Nucleic Acids Res.

[47] Jabbari H, Condon A, Pop A, Zhao Y. HFold: RNA Pseudoknotted Secondary Structure Prediction Using Hierarchical Folding. In: Algorithms in Bioinformatics, pp. 323–334. Springer, Berlin, 2007. 10.1007/978-3-540-74126-8_30.

[48] Witwer C, Hofacker I, Stadler P (2004). Prediction of consensus RNA secondary structures including pseudoknots. IEEE/ACM Trans Comput Biol Bioinform.

[49] Rivas E, Clements J, Eddy SR (2017). A statistical test for conserved RNA structure shows lack of evidence for structure in lncRNAs. Nat Methods.

[50] Rivas E, Clements J, Eddy SR (2020). Estimating the power of sequence covariation for detecting conserved RNA structure. Bioinformatics.

[51] Rivas E, Lang R, Eddy SR (2012). A range of complex probabilistic models for RNA secondary structure prediction that includes the nearest-neighbor model and more. RNA.

[52] Knudsen B, Hein J (1999). RNA secondary structure prediction using stochastic context-free grammars and evolutionary history. Bioinformatics.

[53] Dowell RD, Eddy SR (2004). Evaluation of several lightweight stochastic context-free grammars for RNA secondary structure prediction. BMC Bioinform.

[54] Sloma MF, Mathews DH (2016). Exact calculation of loop formation probability identifies folding motifs in RNA secondary structures. RNA.

[55] Huang L, Zhang H, Deng D, Zhao K, Liu K, Hendrix DA, Mathews DH (2019). Linearfold: linear-time approximate RNA folding by 5’-to-3’ dynamic programming and beam search. Bioinformatics.

[56] Kalvari I, Nawrocki EP, Ontiveros-Palacios N, Argasinska J, Lamkiewicz K, Marz M, Griffiths-Jones S, Toffano-Nioche C, Gautheret D, Weinberg Z, Rivas E, Eddy SR, Finn RD, Bateman A, Petrov AI (2021). Rfam 14: expanded coverage of metagenomic, viral and microRNA families. Nucleic Acids Res.

[57] Pak D, Root-Bernstein R, Burton ZF (2017). tRNA structure and evolution and standardization to the three nucleotide genetic code. Transcription.

[58] de Lencastre A, Pyle AM (2008). Three essential and conserved regions of the group II intron are proximal to the 5‘-splice site. RNA.

[59] Nawrocki EP, Jones TA, Eddy SR (2018). Group I introns are widespread in archaea. Nucleic Acids Res.

[60] Wright ES (2020). RNAconTest: comparing tools for non-coding RNA multiple sequence alignment based on structural consistency. RNA.

[61] Edgar RC (2004). MUSCLE: a multiple sequence alignment method with reduced time and space complexity. BMC Bioinform.

[62] Katoh K, Standley DM (2013). MAFFT multiple sequence alignment software version 7: improvements in performance and usability. Mol Biol Evol.

[63] Hajiaghayi M, Condon A, Hoos HH (2012). Analysis of energy-based algorithms for RNA secondary structure prediction. BMC Bioinform.

[64] Rivas E (2021). Evolutionary conservation of RNA sequence and structure. WIREs RNA.

[65] Darty K, Denise A, Ponty Y (2009). VARNA: interactive drawing and editing of the RNA secondary structure. Bioinformatics.

[66] Vierna J, Wehner S, zu Siederdissen CH, Martínez-Lage A, Marz M (2013). Systematic analysis and evolution of 5S ribosomal DNA in metazoans. Heredity.

[67] Martinez-Porchas M, Villalpando-Canchola E, Suarez LEO, Vargas-Albores F (2017). How conserved are the conserved 16S-rRNA regions?. Heredity.

[68] Peker N, Garcia-Croes S, Dijkhuizen B, Wiersma HH, van Zanten E, Wisselink G, Friedrich AW, Kooistra-Smid M, Sinha B, Rossen JWA, Couto N (2019). A comparison of three different bioinformatics analyses of the 16S–23S rRNA encoding region for bacterial identification. Front Microbiol.

[69] Bernier CR, Petrov AS, Kovacs NA, Penev PI, Williams LD (2018). Translation: the universal structural core of life. Mol Biol Evol.

[70] Haas ES, Brown JW (1998). Evolutionary variation in bacterial RNase P RNAs. Nucleic Acids Res.

[71] Andersen ES, Rosenblad MA, Larsen N, Westergaard JC, Burks J, Wower IK, Wower J, Gorodkin J, Samuelsson T, Zwieb C (2006). The tmRDB and SRPDB resources. Nucleic Acids Res.

[72] Gunisova S, Elboher E, Nosek J, Gorkovoy V, Brown Y, Lucier J, Laterreur N, Wellinger RJ, Tzfati Y, Tomaska L (2009). Identification and comparative analysis of telomerase RNAs from Candida species reveal conservation of functional elements. RNA.

[73] Zwieb C, Wower I, Wower J (1999). Comparative sequence analysis of tmRNA. Nucleic Acids Res.

