# Supplementary material for: KnotAli: informed energy minimization through the use of evolutionary information
Source: BMC Bioinformatics. 2022 May 3;23:159. doi: 10.1186/s12859-022-04673-3 (PMC9063079; doi:10.1186/s12859-022-04673-3)
Supplement: Supplementary file 1 — Additional file 1. Recurrences. We provide the recurrences which make up the thermodynamic MFE prediction within KnotAli. [file 12859_2022_4673_MOESM1_ESM.pdf]

Supplementary File 1: Recurrences

February 17, 2022

These recurrences were previously presented in [1] and [2]. We start by giving any notation used within this supplementary file and follow with the recurrences.

1 Notation

The following notations, used within this supplementary file, is outlined in this section. We let G denote a pseudoknot-free guide structure.

- $bp(i)$ is used to denote the base which pairs with i within the guide structure G.
- *Cover*: Assume k is an index in the guide structure G; k is considered covered if there is a base pair $i.j$ where $i < k < j$ and there is other base pair $i'.j'$ such that $i < i' < k < j' < j$.
- $isCovered(G, i)$ is true if and only if the base is is covered within the guide structure G.

2 Recurrences

$W(i, j)$ holds the energy of the MFE pseudoknotted secondary structure for region $[i, j]$ of the structure over sequence S.

$$W(i,j) = \min \begin{cases} W(i, j-1) \\ V(i,j) \\ \min_{i \leq k < j} W(i,k) + W(k+1, j) \text{ k is not covered} \\ WMB(i,j) + P_s \end{cases} \tag{1}$$

$WI(i, j)$ holds the energy of the MFE pseudoknotted secondary structure for region $[i, j]$ when the region is inside a pseudoknot.

$$WI(i,j) = \min \begin{cases} V(i,j) + P_{ps} \text{ if } i.j \in G \text{ or both are unpaired in } G \\ \min\limits_{i \leq k < j} WI(i,k) + WI(k+1, j) \\ WMB(i,j) + P_{sm} + P_{ps} \end{cases} \tag{2}$$

$WI'(i, j)$ holds the energy of the MFE pseudoknotted secondary structure for region $[i, j]$ when the region is inside a band.

$$WI'(i,j) = \min \begin{cases} V(i,j) + b' \text{ if } i.j \in G \text{ or both are unpaired in } G \\ WI'(i+1, j) + c' \text{ if } bp(i) = 0 \\ WI'(i, j-1) + c' \text{ if } bp(j) = 0 \\ \min\limits_{i \leq k < j} WI'(i,k) + WI'(k+1, j) \\ WMB(i,j) + P_{sm} + b' \end{cases} \tag{3}$$

$VP(i,j)$ holds the energy of the MFE pseudoknotted secondary structure for region $[i,j]$ closed by predicted base pair $i.j$ which crosses a base pair in G.

$$VP(i,j) = \min \begin{cases} WI(i+1, B'(i,j)-1) + WI(B(i,j)+1, j-1) \text{ if } isCovered(G,i) \text{ and NOT } iscovered(G,j), \\ WI(i+1, b(i,j)-1) + WI(b'(i,j)+1, j-1) \text{ if NOT } isCovered(G,i) \text{ and } iscovered(G,j), \\ WI(i+1, B'(i,j)-1) + WI(B(i,j)+1, b(i,j)-1) + WI(b'(i,j)+1, j-1) \\ \text{ if } isCovered(G,i) \text{ and } iscovered(G,j), \\ e_{stP}(i, i+1, j-1, j) + VP(i+1, j-1) \text{ if } bp(i+1) = bp(j-1) = 0, \\ \min\limits_{i \le k < min(B'(i,j), b(i,j))} e_{intP}(i,k,k'j) \\ \min\limits_{i \le k < min(B'(i,j), j)} WI'(i+1, k-1) + VP'(k, j-1) + a' + 2b' \\ \min\limits_{max(i, b'(i,j)) \le k < j} VP'(i+1, k) + WI'(k+1, j-1) + a' + 2b' \end{cases} \tag{4}$$

$VP'(i,j)$ holds the energy of the MFE pseudoknotted secondary structure for region $[i,j]$ where within a predicted base pair $i.r$ or $r.j$ for some r exists where $i < r < j$ and $i.r$ or $r.j$ crosses a base pair in G.

$$VP'(i,j) = \min \begin{cases} \min\limits_{max(i, b'(i,j)) \le k < j} VP(i,k) + WI'(k+1, j) \\ \min\limits_{i \le k < min(B'(i,j), j)} WI'(i, k-1) + VP(k, j) \\ \min\limits_{max(i, b'(i,j)) \le k < j} VP(i,k) + c'(j-k) \\ \min\limits_{i \le k < min(B'(i,j), j)} c'(k-i) + VP(k,j) \end{cases} \tag{5}$$

$V(i,j)$ holds the energy of the MFE pseudoknotted secondary structure for region $[i,j]$ closed by the base pair $i.j$.

$$V(i,j) = \min \begin{cases} e_H(i,j) \\ e_s(i,j) + V(i+1, j-1) \\ VBI(i,j) \\ VM(i,j) \end{cases} \tag{6}$$

$VBI(i,j)$ holds the energy of the MFE pseudoknotted secondary structure for region $[i,j]$ where $i.j$ and $i.j$ closes a bulge or internal loop.

$$VBI(i,j) = \begin{cases} \min\limits_{i<k<k'<j} e_{int}(i,k,k',j) + V(k,k') \end{cases} \tag{7}$$

$VM(i,j)$ holds the energy of the MFE pseudoknotted secondary structure for region $[i,j]$ where $i.j$ and $i.j$ closes a multiloop,

$$VM(i,j) = \min \begin{cases} \min\limits_{i+1<k \le j-1} WM(i+1, k-1) + WM(k, j-1) + a + b \\ WMB(i+1, j-1) + a + P_{sm} + b \end{cases} \tag{8}$$

$WM(i,j)$ holds the energy of the MFE pseudoknotted secondary structure for region $[i,j]$ where a base pair exists within i and j, and i and j are on a multibranched loop.

$$WM(i,j) = \min \begin{cases} V(i,j) + b \\ WM(i+1, j) + c \\ WM(i, j-1) + c \\ \min\limits_{i \le k \le j-1} WM(i,k) + WM(k+1, j) \\ WMB(i+1, j-1) + P_{sm} + b \end{cases} \tag{9}$$

$WMB(i,j)$ holds the energy of the MFE pseudoknotted secondary structure for region $[i,j]$ where within i and j exists a density-2 pseudoloop

$$WMB(i,j) = \min \begin{cases} P_b + \min\limits_{bp(j)<k<j} BE(bp(j), bp(B'(k,j)), B'(k,j), j) \\ \qquad + WMB'(i,k) + WI(k+1, B'(k,j)-1) \\ WMB'(i,j) \end{cases} \tag{10}$$

$WMB'(i,j)$ holds the energy of the MFE pseudoknotted secondary structure for region $[i,j]$ where within i and j, i starts the first band of the pseudoloop and j either closes the pseudoloop starting at i, is the closing base of a region within the pseudoloop starting at i, is an unpaired base outside the first two bands, or a closing base of a band associated with the pseudoloop.

$$WMB'(i,j) = \min \begin{cases} 2P_b + \min\limits_{i<k<min(j,b(i,j)} BE(bp(B(k,j)), bp(B'(k,j)), B'(k,j), B(k,j) \\ \qquad + WMB'(i,k-1) + VP(k,j) \\ \min\limits_{i<k<j} WMB'(i,k) + WI(k+1,j) \\ P_b + VP(i,j) \\ 2P_b + \min\limits_{i<k<bp(i)} BE(i, b'(i,k), bp(b'(i,k), bp(i)) + WI(b'(i,k), k-1) \\ \qquad + VP(k,j) \end{cases} \tag{11}$$

$BE(i,k,k',j)$ holds the energy of the MFE pseudoknotted secondary structure for the band $[i,k] \cup [k',j]$ where $i \leq k < k' \leq j$ and $i.j$ and $k.k'$.

$$BE(i,k,k',j) = \min \begin{cases} e_{stP}(i,j) + BE(i+1,k,k',j-1) \\ e_{intP}(i,l,bp(l),j) + BE(l,k,k',bp(l)) \\ WI'(i+1,l-1) + BE(l,k,k',bp(l)) + WI'(bp(l)+1,j-1) + a' + 2b' \\ WI'(i+1,l-1) + BE(l,k,k',bp(l)) + a' + 2b' + c'(j-bp(l)+1) \\ a' + 2b' + c'(l-i+1) + BE(l,k,k',bp(l)) + WI'(bp(l)+1,j-1) \end{cases} \tag{12}$$

References

[1] Jabbari, H., Condon, A., Pop, A., Zhao, Y.: HFold: RNA Pseudoknotted Secondary Structure Prediction Using Hierarchical Folding. In: Algorithms in Bioinformatics, pp. 323–334. Springer, Berlin, Heidelberg (2007). doi:10.1007/978-3-540-74126-8_30. https://doi.org/10.1007/978-3-540-74126-8_30

[2] Jabbari, H., Condon, A.: A fast and robust iterative algorithm for prediction of RNA pseudoknotted secondary structures. BMC Bioinformatics **15** (2014). doi:10.1186/1471-2105-15-147

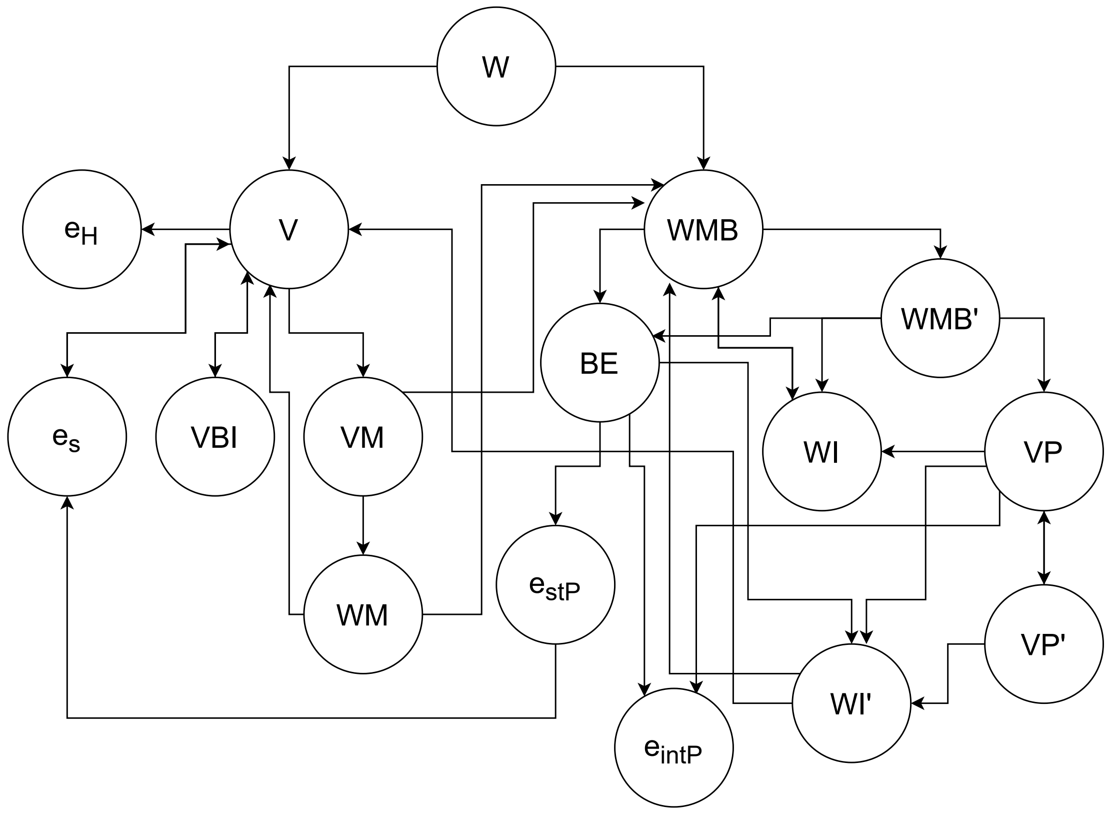

Figure 1: Illustration of the recurrences
