# Supplementary material for: KnotAli: informed energy minimization through the use of evolutionary information
Source: BMC Bioinformatics. 2022 May 3;23:159. doi: 10.1186/s12859-022-04673-3 (PMC9063079; doi:10.1186/s12859-022-04673-3)
Supplement: Supplementary file 2 — Additional file 2. Energy Table. The table shows the free energy parameters which determine the free energy of the structures. [file 12859_2022_4673_MOESM2_ESM.pdf]

Supplementary File 1: Energy Table

December 21, 2021

1 Notation

We give the notation for the parameters which make up the energy model. Values are first given for the parameters used in a pseudoknot-free structure and then move to those within a pseudoknotted.

Name	Description	Energy ($KCal/mol$)
$e_H(i,j)$	Energy of a hairpin closed by $i.j$	
$e_S(i,j)$	Energy of a stack closed by $i.j$	
$e_{stP}(i,j)$	Energy of a stack that spans a band	$.89 \cdot e_S(i,j)$
$e_{int}(i,k,l,j)$	Energy of a pseudoknot-free internal loop	
$e_{intP}(i,k,l,j)$	Energy of a internal loop that spans a band	$.74 \cdot e_{int}(i,k,l,j)$
P_s	Exterior pseudoloop initiation penalty	-1.38
P_{sm}	Penalty for initiation of pseudoloop in a multiloop	10.07
P_{sp}	Penalty for initiation of pseudoloop in a pseudoloop	15.0
P_b	Penalty for initiating a band	2.46
P_{up}	Penalty for unpaired base of a pseudoloop	.06
P_{ps}	Penalty for closed subregion	.96
a	Penalty for initiation of a multiloop	3.36
b	multiloop base pair penalty	.03
c	Penalty for unpaired base of a multiloop	.02
a'	Penalty for initiation of a multiloop that spans a band	3.41
b'	Branch penalty in a multiloop that spans a band	.56
c'	Penalty for unpaired base in a multiloop that spans a band	.12

Table 1: Free energy parameters used within the thermodynamic portion of our algorithm. The table shows the name of the parameter, its description, and its corresponding energy value. The values are taken from the parameters from HotKnots V2 [1]. All values were found at a temperature of 37°C and with 1 M salt concentration

References

[1] Andronescu, M.S., Pop, C., Condon, A.E.: Improved free energy parameters for RNA pseudoknotted secondary structure prediction. RNA **16**, 26–42 (2010). doi:10.1261/rna.1689910
