# Supplementary material for: KnotAli: informed energy minimization through the use of evolutionary information
Source: BMC Bioinformatics. 2022 May 3;23:159. doi: 10.1186/s12859-022-04673-3 (PMC9063079; doi:10.1186/s12859-022-04673-3)
Supplement: Supplementary file 3 — Additional file 3. P-tables. We provide all non-included tables from our paper. Section 1 gives the MI vs MIp comparison table and the associated p-values. Section 2 gives the Restricted vs Non-restricted table and its associated p-values. Section 3 gives the non-included heatmaps for MUSCLE. Section 4 gives the p-values for the comparison with existing algorithms tables included in the text. Section 5 gives the p-values for varying sequence length table. Section 6 gives the p-values for when compatible bases are not considered false positives. Section compares the results between the other algorithms and against themselves. [file 12859_2022_4673_MOESM3_ESM.pdf]

Supplementary File 3: P-tables

1 MI vs MIp

	MUSCLE						MAFFT					
	MI			MIp			MI			MIp		
family	sen	ppv	F	sen	ppv	F	sen	ppv	F	sen	ppv	F
5s	.269	.950	.419	.625	.894	**.735**	.148	.983	.252	.612	.897	**.726**
16s	.107	.836	.189	.268	.648	**.378**	.125	.906	.220	.315	.744	**.441**
23s	.041	.472	.077	.110	.431	**.175**	.048	.504	.087	.107	.395	**.169**
Group I Intron	0	0	0	.021	.478	**.040**	.006	.629	.012	.059	.463	**.104**
Group II Intron	.003	.031	.006	.005	.012	.007	.015	.146	.027	.047	.099	**.062**
RNaseP	0	0	0	.115	.658	**.193**	.032	.661	.061	.225	.747	**.338**
SRP	.049	.889	.089	.156	.780	**.238**	.078	.885	.135	.126	.627	**.202**
telomerase	0	0	0	.025	.205	**.044**	0	0	0	.018	.185	**.033**
tmRNA	.049	.983	.093	.130	.917	**.227**	.048	.978	.091	.151	.785	**.252**
tRNA	.673	.972	.793	.810	.973	**.881**	.547	.930	.684	.743	.927	**.819**

Table 1: Mean F-measures for KnotAli when MI is used as the covariation metric versus the results of MIp which are the standard KnotAli covariation measure prior to the thermodynamic portion. Each column represents the mean F-measure for one of the covariation metrics.**BOLD** is used to show significant difference in the results.

family	MUSCLE	MAFFT
5s	.00009	.00009
16s	.00009	.00009
23s	.014	.014
Group I Intron	.00009	.00009
Group II Intron	.789	.00009
RNaseP	.00009	.00009
SRP	.00009	.00009
telomerase	.00009	.00009
tmRNA	.00009	.00009
tRNA	.00009	.00009

Table 2: P-values for KnotAli when MI is used as the covariation metric versus the results of MIp which are the standard KnotAli covariation measure. Each column represents the p-value for one of the covariation metrics.

2 Restricted vs non-restricted

	MUSCLE		MAFFT	
family	Non-Restricted	Restricted	Non-Restricted	Restricted
5s	.850	**.887**	.803	**.885**
16s	.515	.494	.523	.519
Group I Intron	.461	.461	.435	.396
Group II Intron	.154	.154	.258	.295
RNaseP	.482	.493	.539	**.585**
SRP	.507	**.564**	.390	.415
telomerase	**.368**	.256	.235	.211
tmRNA	.366	**.477**	.495	.495
tRNA	.859	**.932**	.840	**.886**

Table 3: Mean F-measures for KnotAli when restricted bases are not used in KnotAli versus when they are, which is the standard for KnotAli. Each column represents the mean F-measure.**BOLD** is used to show significant difference in the results.

family	MUSCLE	MAFFT
5s	.00009	.00009
16s	.803	.953
Group I Intron	.982	.214
Group II Intron	.816	.582
RNaseP	.509	.002
SRP	.0003	.105
telomerase	.003	.530
tmRNA	.00009	1
tRNA	.00009	.0003

Table 4: P-values for KnotAli when restricted bases are not used in KnotAli versus when they are, which is the standard for KnotAli. Each column represents the p-values.

3 Gridsearch

thresh	tRNA	5s	SRP	tmRNA	RNaseP	16s	23s	Group I Intr	Group II Intr	telomerase
-0.2	.828	.805	.290	.289	.446	.486	.208	.176	.083	.057
-0.1	.828	.805	.290	.289	.446	.490	.203	.177	.083	.057
0	.830	.808	.291	.288	.446	.466	.196	.177	.084	.048
0.1	.849	.788	.267	.299	.450	.462	.191	.169	.088	.051
0.2	.845	.763	.260	.299	.423	.463	.184	.168	.082	.028
0.3	.819	.729	.251	.270	.386	.454	.181	.140	.068	.030
0.4	.819	.726	.202	.252	.338	.442	.169	.104	.063	.033
0.5	.819	.677	.177	.214	.296	.401	.161	.044	.056	.018
0.6	.819	.639	.161	.185	.253	.351	.148	.026	.038	.018
0.7	.744	.592	.135	.136	.180	.297	.127	.012	.027	.018
0.8	.713	.524	.135	.108	.106	.251	.113	.012	.028	0
0.9	.713	.375	.135	.091	.075	.214	.088	.012	.029	0
1	.684	.207	.094	.073	.013	.186	.074	.012	.030	0
1.1	.651	.165	.094	.057	0	.141	.048	.012	.030	0
1.2	.651	.165	.094	.038	0	.097	.014	0	.030	0
1.3	.489	.165	.049	.038	0	.065	.005	0	.016	0
1.4	.155	.165	0	.020	0	.045	.002	0	0	0
1.5	.008	0	0	.020	0	.013	0	0	0	0

Table 5: The heatmap illustrates the results of a grid search across 21 different possible thresholds on the 10 families. The values of the heatmap represent the mean F-measure for the family at the specific threshold using the MUSCLE aligner.

thresh	tRNA	5s	SRP	tmRNA	RNaseP	16s	23s	Group I In	Group II Intr	telomerase
-0.2	.811	.81	.455	.395	.540	.569	.29	.244	.087	.074
-0.1	.811	.81	.455	.395	.540	.573	.282	.246	.087	.074
0	.815	.817	.459	.400	.542	.576	.359	.249	.090	.069
0.1	.903	.867	.453	.486	.570	.600	.365	.262	.098	.088
0.2	.925	.885	.500	.637	.609	.654	.372	.284	.099	.064
0.3	.927	.885	.553	.700	.682	.683	.383	.362	.091	.081
0.4	.927	.897	.627	.785	.747	.744	.395	.463	.099	.185
0.5	.927	.947	.818	.854	.775	.792	.421	.573	.115	.192
0.6	.927	.970	.837	.897	.766	.834	.443	.537	.108	.247
0.7	.930	.982	.885	.951	.730	.898	.551	.629	.113	.608
0.8	.929	.983	.885	.975	.758	.926	.555	.629	.168	0
0.9	.929	.985	.885	.978	.764	.943	.648	.629	.302	0
1	.930	.988	.885	.974	.634	.945	.655	.629	.818	0
1.1	.928	.995	.885	.974	0	.956	.703	.629	.818	0
1.2	.928	.995	.885	.963	0	.965	.644	0	1	0
1.3	.920	.995	.871	.963	0	.973	.400	0	1	0
1.4	.885	.995	0	.972	0	.974	1	0	0	0
1.5	.879	.008	0	.972	0	.947	0	0	0	0

Table 6: The heatmap illustrates the results of a grid search across 21 different possible thresholds on the 10 families. The values of the heatmap represent the mean PPV for the family at the specific threshold using the MUSCLE aligner.

4 Comparison with existing algorithms

family	Knot to RNA	Knot to Hex	Knot to Cac	RNA to Hex	RNA to Cac	Hex to Cac
5s	.00009	.00009	.00009	.00009	.00009	.00009
16s	.030	.838	.00009	.011	.00009	.00009
23s	.014	.145	.014	.014	.014	.014
Group I Intron	.00009	.00009	.00009	.020	.040	.674
Group II Intron	.00009	.003	.00009	.211	1	.211
RNaseP	.00009	.00009	.00009	.00009	.00009	.021
SRP	.00009	.00009	.00009	.521	.291	.680
telomerase	.00009	.00009	.00009	.00009	.00009	.409
tmRNA	.00009	.00009	.00009	.00009	.0003	.00009
tRNA	.059	.00009	.447	.00009	.007	.00009

Table 7: P-values for KnotAli, RNAalifold, Hxmatch, and Cacofold. Each column represents the p-value for one of the algorithms when compared to one of the other algorithms. These are for when the input alignment are through MUSCLE Abbreviations are made for each algorithm: Knot (KnotAli), RNA (RNAalifold), Hex (Hxmatch), and Cac (Cacofold).

family	Knot to RNA	Knot to Hex	Knot to Cac	RNA to Hex	RNA to Cac	Hex to Cac
5s	.00009	.00009	.00009	.00009	.00009	.00009
16s	.007	.225	.00009	.006	.00009	.00009
23s	.014	.014	.430	.014	.014	.014
Group I Intron	.00009	.00009	.00009	1	.019	.019
Group II Intron	.021	.008	.012	.373	.891	.286
RNaseP	.002	.00009	.00009	.00009	.00009	.00009
SRP	.00009	.00009	.00009	.453	.00009	.00009
telomerase	.0004	.00009	.00009	.004	.00009	.00009
tmRNA	.00009	.00009	.00009	.00009	.00009	.00009
tRNA	.017	.00009	.753	.00009	.056	.00009

Table 8: P-values for KnotAli, RNAalifold, Hxmatch, and Cacofold. Each column represents the p-value for one of the algorithms when compared to one of the other algorithms. These are for when the input alignment are through MAFFT Abbreviations are made for each algorithm: Knot (KnotAli), RNA (RNAalifold), Hex (Hxmatch), and Cac (Cacofold).

5 Varying sequence length in alignment

We restricted the length of two families within our dataset: Group I Intronand SRP. The number of sequences for each family was decreased to 33 and 285, respectively.

When comparing post-shortened results to pre-shortened results, the 33 of the total 89 and 285 of the total 583 sequences were compared for pre-shortened corresponding to the sequences in post-shortened.

family	Knot to RNA	Knot to Hex	Knot to Cac	RNA to Hex	RNA to Cac	Hex to Cac
Group I Intron-MAFFT	.00009	.00009	.00009	.0002	.00009	.00009
SRP-MAFFT	.00009	.00009	.00009	.1482	.00009	.00009
Group I Intron-MUSCLE	.00009	.00009	.00009	.016	.137	.136
SRP-MUSCLE	.00009	.00009	.00009	.0002	.00009	.00009

Table 9: P-values for KnotAli, RNAalifold, Hxmatch, and Cacofold when the range of length for sequences is shortened. Each column represents the p-value for one of the algorithms when compared to one of the other algorithms. These are for when the input alignment are through MAFFT and MUSCLE. Abbreviations are made for each algorithm: Knot (KnotAli), RNA (RNAalifold), Hex (Hxmatch), and Cac (Cacofold).

family	KnotAli	RNAalifold	Hxmatch	Cacofold
Group I Intron-MAFFT	.488	.078	.078	.122
SRP-MAFFT	.391	.056	.057	.125
Group I Intron-MUSCLE	.509	.119	.080	.080
SRP-MUSCLE	.448	.101	.102	.102

Table 10: Mean F-measures for KnotAli, RNAalifold, Hxmatch, and Cacofold in pre-shortened when only the sequences seen in post-shortened are looked at. Each column represents the mean F-measure for one of the algorithms.

6 Accuracy measures

family	KnotAli	RNAalifold	Hxmatch	Cacofold
Group I Intron-MAFFT	.229	.00009	.00009	.427
SRP-MAFFT	.00009	.00009	.00009	.00009
Group I Intron-MUSCLE	.341	.0009	.001	.00009
SRP-MUSCLE	.023	.00009	.00009	.00009

Table 11: P-values for KnotAli, RNAalifold, Hxmatch, and Cacofold when the range of length for sequences is shortened. Each column represents the p-value for one of the algorithms when compared to its original non-shortened value.

family	Knot to RNA	Knot to Hex	Knot to Cac	RNA to Hex	RNA to Cac	Hex to Cac
5s	.00009	.00009	.00009	.00009	.00009	.00009
16s	.094	.495	.00009	.005	.00009	.00009
23s	.014	.324	.014	.014	.014	.014
Group I Intron	.00009	.00009	.00009	.020	.089	.462
Group II Intron	.00009	.0007	.0009	.212	1	.211
RNaseP	.00009	.00009	.00009	.00009	.00009	.00009
SRP	.00009	.00009	.00009	.999	.267	.267
telomerase	.00009	.009	.0003	.00009	.00009	.003
tmRNA	.00009	.00009	.00009	.00009	.0004	.00009
tRNA	.256	.00009	.00009	.00009	.006	.00009

Table 12: P-values for KnotAli, RNAalifold, Hxmatch, and Cacofold when compatible bases are not considered as false positive. Each column represents the p-value for one of the algorithms when compared to one of the other algorithms. These are for when the input alignment are through MUSCLE Abbreviations are made for each algorithm: Knot (KnotAli), RNA (RNAalifold), Hex (Hxmatch), and Cac (Cacofold).

family	Knot to RNA	Knot to Hex	Knot to Cac	RNA to Hex	RNA to Cac	Hex to Cac
5s	.00009	.00009	.00009	.00009	.00009	.00009
16s	.005	.437	.00009	.002	.00009	.00009
23s	.014	.043	.430	.014	.014	.014
Group I Intron	.00009	.00009	.00009	1	.009	.009
Group II Intron	.00009	.00009	.0002	.600	.870	.600
RNaseP	.00009	.00009	.00009	.00009	.00009	.00009
SRP	.00009	.00009	.00009	1	.00009	.00009
telomerase	.010	.006	.00009	.008	.00009	.00009
tmRNA	.00009	.00009	.00009	.00009	.00009	.00009
tRNA	.00009	.00009	.032	.00009	.043	.00009

Table 13: P-values for KnotAli, RNAalifold, Hxmatch, and Cacofold when compatible bases are not considered as false positive. Each column represents the p-value for one of the algorithms when compared to one of the other algorithms. These are for when the input alignment are through MAFFT Abbreviations are made for each algorithm: Knot (KnotAli), RNA (RNAalifold), Hex (Hxmatch), and Cac (Cacofold).

family	KnotAli	RNAalifold	Hxmatch	Cacofold
5s	.00009	.00009	.440	.00009
16s	.224	.388	.471	.136
23s	.041	.014	.014	.014
Group I Intron	.005	.896	.874	.863
Group II Intron	.604	1	.816	1
RNaseP	.005	.672	.994	.747
SRP	.0006	.520	.998	.491
telomerase	.074	.217	.282	.002
tmRNA	.00009	.113	.640	.355
tRNA	.002	.870	.897	.847

Table 14: P-values for KnotAli, RNAalifold, Hxmatch, and Cacofold when compatible bases are not considered as false positive. Each column represents the p-value for one of the algorithms compared in this work. These are for when the input alignment are through MUSCLE

family	KnotAli	RNAalifold	Hxmatch	Cacofold
5s	.00009	.00009	.017	.00009
16s	.425	.279	.426	.112
23s	.517	.014	.014	.014
Group I Intron	.0002	1	1	.779
Group II Intron	.093	.930	.586	.891
RNaseP	.00009	.9425	.873	.401
SRP	.002	.452	1	.215
telomerase	.079	.004	.075	.084
tmRNA	.00009	.268	.567	.090
tRNA	.009	.866	.899	.818

Table 15: P-values for KnotAli, RNAalifold, Hxmatch, and Cacofold when compatible bases are not considered as false positive. Each column represents the p-value for one of the algorithms when compared to its result where compatible bases are considered false positives. These are for when the input alignment are through MAFFT
