# Supplementary material for: KnotAli: informed energy minimization through the use of evolutionary information
Source: BMC Bioinformatics. 2022 May 3;23:159. doi: 10.1186/s12859-022-04673-3 (PMC9063079; doi:10.1186/s12859-022-04673-3)
Supplement: Supplementary file 4 — Additional file 4. Cross-validation. We provide our validation results for the heatmap-based threshold pick. Validation results were done through a 70-30 split over 1000 iterations where the threshold was picked based on the 70% training set and assessed on the 30% test set. [file 12859_2022_4673_MOESM4_ESM.pdf]

Supplementary File 4: Cross-validation

1 70-30 data split

Validation was performed on the results of the grid search (see Section 4.2 of the manuscript). Note that the grid search was performed on base pair information obtained using the adjusted mutual information (`MIp`) before the thermodynamic prediction. We aimed to choose the highest threshold level with acceptable F-measure. We performed 1000 iterations of randomly splitting the data in a 70/30 split, finding the mean of all iterations for both splits. This was performed for both the MAFFT and MUSCLE aligner for the F-measure and the PPV. We refer to the 70% of the data as training set and the 30% as test set. We chose the threshold based on the training set and assessed its fit using the test set. Following, we present 4 tables corresponding to F-measure and PPV for both training set and test set for MUSCLE (Section 1.1) and MAFFT (Section 1.2).

1.1 MUSCLE

The following heatmaps present changes in F-measure values (see Table 1 and Table 3) and PPV values (see Table 2 and Table 4) in the training set and test set when the sequence alignment was generated using MUSCLE. Note that threshold value of 0.4 represents the highest threshold value after which both F-measure and PPV see significant change at least in one family.

thresh	tRNA	5s	SRP	tmRNA	RNaseP	16s	23s	GI	GII	telomerase
-0.2	.838	.42	.207	.125	.016	.379	.241	.101	.337	.855
-0.1	.838	.422	.196	.125	.017	.379	.241	.112	.337	.855
0.0	.844	.411	.194	.126	.016	.379	.245	.082	.332	.855
0.1	.835	.402	.194	.115	.012	.385	.224	.087	.302	.883
0.2	.794	.402	.185	.1	.01	.364	.231	.05	.309	.889
0.3	.748	.393	.184	.076	.008	.307	.242	.04	.283	.908
0.4	.735	.377	.175	.04	.007	.193	.239	.044	.227	.881
0.5	.735	.354	.166	.027	.006	.187	.223	.017	.161	.882
0.6	.708	.324	.159	.005	.006	.109	.223	.017	.152	.881
0.7	.66	.273	.133	0	.006	.061	.223	.017	.111	.881
0.8	.574	.235	.119	0	0	.049	.165	.017	.111	.854
0.9	.419	.185	.091	0	0	.012	.165	0	.111	.824
1.0	.419	.146	.08	0	0	0	.089	0	.075	.768
1.1	.356	.101	.042	0	0	0	.048	0	.075	.735
1.2	.279	.058	.012	0	0	0	0	0	.059	.7
1.3	.279	.028	.005	0	0	0	0	0	.04	.7
1.4	.102	.014	.002	0	0	0	0	0	.04	.579
1.5	.052	.004	0	0	0	0	0	0	.02	.371

Table 1: Each column consists of the mean F-measure for a family for the specific threshold in the training set obtained from the multiple sequence alignment created using MUSCLE. We observe a decline in F-measure at threshold value of 0.4.

thresh	tRNA	5s	SRP	tmRNA	RNaseP	16s	23s	GI	GII	telomerase
-0.2	.819	.49	.284	.164	.019	.427	.322	.13	.43	.861
-0.1	.819	.49	.275	.164	.02	.427	.322	.143	.43	.861
0.0	.832	.504	.365	.169	.02	.427	.344	.114	.439	.862
0.1	.87	.518	.384	.169	.017	.465	.335	.134	.472	.92
0.2	.877	.55	.391	.204	.015	.554	.402	.101	.628	.932
0.3	.877	.586	.413	.263	.013	.611	.564	.104	.788	.973
0.4	.894	.647	.431	.478	.012	.658	.78	.205	.917	.973
0.5	.933	.705	.446	.486	.014	.66	.897	.14	.947	.973
0.6	.942	.747	.488	.27	.018	.674	.897	.36	.963	.973
0.7	.947	.819	.58	0	.025	.669	.897	.893	.984	.973
0.8	.96	.863	.587	0	0	.669	.899	.891	.984	.973
0.9	.95	.917	.68	0	0	.59	.898	0	.984	.973
1.0	.95	.922	.716	0	0	0	.89	0	.982	.972
1.1	.958	.951	.682	0	0	0	.849	0	.982	.974
1.2	.955	.951	.556	0	0	0	0	0	.984	.976
1.3	.956	.956	.4	0	0	0	0	0	.985	.976
1.4	.927	.977	.0	0	0	0	0	0	.985	.976
1.5	.905	.907	0	0	0	0	0	0	.97	.969

Table 2: Each column consists of the mean PPV for a family for the specific threshold in the training set obtained from the multiple sequence alignment created using MUSCLE. We observe a sharp change in PPV at threshold value of 0.4.

thresh	tRNA	5s	SRP	tmRNA	RNaseP	16s	23s	GI	GII	telomerase
-0.2	.838	.419	.207	.126	.016	.378	.241	.101	.337	.856
-0.1	.838	.422	.196	.126	.016	.378	.241	.112	.337	.855
0.0	.844	.414	.194	.126	.016	.379	.246	.082	.333	.855
0.1	.835	.401	.194	.116	.012	.384	.224	.087	.302	.883
0.2	.794	.401	.185	.1	.01	.364	.232	.049	.309	.889
0.3	.748	.392	.184	.076	.009	.309	.242	.04	.283	.908
0.4	.735	.379	.175	.04	.007	.193	.238	.044	.227	.882
0.5	.735	.353	.166	.027	.006	.188	.223	.017	.161	.881
0.6	.708	.326	.159	.005	.006	.11	.223	.017	.153	.882
0.7	.66	.273	.133	0	.006	.061	.224	.017	.111	.882
0.8	.574	.234	.12	0	0	.05	.165	.018	.111	.855
0.9	.419	.186	.091	0	0	.012	.165	0	.111	.824
1.0	.419	.146	.08	0	0	0	.089	0	.075	.769
1.1	.356	.1	.042	0	0	0	.048	0	.075	.735
1.2	.279	.058	.012	0	0	0	0	0	.059	.7
1.3	.279	.028	.005	0	0	0	0	0	.04	.7
1.4	.102	.014	.002	0	0	0	0	0	.04	.58
1.5	.052	.004	0	0	0	0	0	0	.02	.371

Table 3: Each column consists of the mean F-measure for a family for the specific threshold in the test set obtained from the multiple sequence alignment created using MUSCLE. Similar trend of F-measure decline at threshold value of 0.4 is observed here.

1.2 MAFFT

Similar to the previous section, the following heatmaps present changes in F-measure values (see Table 5, and Table 7) and PPV values (see Table 6 and Table 8) in the training set and test set this time using MAFFT as aligner. Note that threshold value of 0.4 represents the highest threshold value after which both F-measure and PPV see significant change at least in one family.

thresh	tRNA	5s	SRP	tmRNA	RNaseP	16s	23s	GI	GII	telomerase
-0.2	.819	.487	.284	.165	.019	.426	.322	.131	.429	.862
-0.1	.819	.49	.275	.165	.019	.426	.322	.143	.43	.861
0.0	.832	.508	.365	.169	.02	.427	.345	.114	.44	.861
0.1	.87	.517	.384	.17	.016	.464	.335	.135	.472	.919
0.2	.877	.548	.391	.204	.014	.555	.402	.1	.628	.931
0.3	.877	.586	.413	.264	.014	.614	.565	.104	.788	.972
0.4	.894	.65	.431	.479	.012	.659	.779	.205	.916	.973
0.5	.932	.704	.446	.486	.014	.662	.896	.14	.947	.973
0.6	.942	.75	.489	.269	.018	.674	.896	.362	.963	.973
0.7	.947	.818	.58	0	.026	.669	.897	.889	.984	.974
0.8	.961	.86	.588	0	0	.671	.898	.893	.984	.974
0.9	.95	.916	.68	0	0	.59	.899	0	.984	.973
1.0	.95	.921	.716	0	0	0	.889	0	.982	.972
1.1	.958	.95	.682	0	0	0	.849	0	.982	.974
1.2	.956	.951	.556	0	0	0	0	0	.984	.976
1.3	.956	.959	.4	0	0	0	0	0	.985	.975
1.4	.926	.979	.0	0	0	0	0	0	.985	.976
1.5	.905	.913	0	0	0	0	0	0	.969	.97

Table 4: Each column consists of the mean PPV for a family for the specific threshold in the test set obtained from the multiple sequence alignment created using MUSCLE. Similar trend of PPV change in at threshold value of 0.4 is observed here.

thresh	tRNA	5s	SRP	tmRNA	RNaseP	16s	23s	GI	GII	telomerase
-0.2	.805	.486	.208	.176	.083	.446	.29	.057	.289	.828
-0.1	.805	.49	.203	.177	.083	.446	.29	.057	.289	.828
0.0	.808	.466	.196	.178	.084	.446	.291	.048	.288	.83
0.1	.789	.462	.191	.179	.088	.45	.267	.051	.299	.849
0.2	.763	.463	.184	.168	.082	.423	.26	.028	.299	.844
0.3	.729	.453	.181	.14	.068	.386	.251	.029	.271	.819
0.4	.726	.441	.169	.104	.063	.339	.202	.033	.252	.82
0.5	.677	.401	.161	.044	.056	.296	.177	.018	.214	.819
0.6	.639	.35	.147	.026	.038	.253	.161	.018	.185	.819
0.7	.592	.297	.127	.012	.027	.18	.135	.018	.136	.744
0.8	.524	.251	.113	.012	.028	.106	.135	0	.108	.713
0.9	.375	.214	.088	.012	.029	.075	.135	0	.091	.713
1.0	.208	.186	.074	.012	.03	.013	.094	0	.073	.684
1.1	.165	.141	.048	.012	.03	0	.094	0	.057	.651
1.2	.165	.097	.014	0	.03	0	.094	0	.038	.651
1.3	.165	.065	.005	0	.016	0	.049	0	.038	.489
1.4	.165	.045	.002	0	0	0	0	0	.02	.155
1.5	.009	.013	0	0	0	0	0	0	.02	.082

Table 5: Each column consists of the mean F-measure for a family for the specific threshold in the training set obtained from the multiple sequence alignment created using MAFFT. We observe a decline in F-measure at threshold value of 0.4.

thresh	tRNA	5s	SRP	tmRNA	RNaseP	16s	23s	GI	GII	telomerase
-0.2	.81	.569	.29	.244	.087	.54	.455	.074	.395	.811
-0.1	.81	.572	.282	.246	.087	.54	.455	.074	.395	.811
0.0	.817	.576	.359	.25	.09	.541	.459	.069	.4	.815
0.1	.867	.601	.365	.262	.098	.57	.452	.088	.486	.904
0.2	.886	.654	.372	.284	.099	.609	.5	.064	.637	.925
0.3	.885	.682	.383	.363	.091	.682	.552	.081	.7	.927
0.4	.897	.743	.395	.463	.099	.748	.627	.185	.785	.927
0.5	.947	.792	.421	.572	.115	.775	.818	.192	.854	.927
0.6	.97	.833	.443	.536	.108	.767	.836	.247	.897	.927
0.7	.982	.897	.551	.629	.112	.73	.885	.61	.951	.929
0.8	.983	.925	.556	.63	.168	.759	.885	0	.975	.928
0.9	.985	.943	.648	.63	.302	.764	.885	0	.978	.928
1.0	.988	.945	.655	.629	.818	.634	.885	0	.974	.93
1.1	.996	.956	.704	.63	.821	0	.885	0	.974	.928
1.2	.996	.965	.644	0	.0	0	.885	0	.963	.928
1.3	.996	.973	.4	0	.0	0	.871	0	.963	.92
1.4	.996	.975	.0	0	0	0	0	0	.973	.885
1.5	.009	.947	0	0	0	0	0	0	.972	.878

Table 6: Each column consists of the mean PPV for a family for the specific threshold in the training set obtained from the multiple sequence alignment created using MAFFT. We observe a sharp change in PPV at threshold value of 0.4.

thresh	tRNA	5s	SRP	tmRNA	RNaseP	16s	23s	GI	GII	telomerase
-0.2	.805	.485	.208	.176	.083	.447	.29	.057	.289	.828
-0.1	.805	.492	.203	.177	.083	.446	.29	.057	.288	.828
0.0	.808	.466	.196	.177	.084	.446	.291	.048	.288	.83
0.1	.789	.461	.191	.18	.088	.451	.267	.052	.299	.849
0.2	.763	.462	.184	.168	.082	.423	.26	.028	.3	.845
0.3	.729	.456	.181	.139	.068	.385	.252	.03	.27	.819
0.4	.726	.443	.169	.104	.063	.337	.202	.033	.252	.819
0.5	.677	.4	.161	.044	.055	.296	.177	.018	.214	.819
0.6	.639	.351	.148	.027	.038	.252	.161	.018	.185	.819
0.7	.592	.298	.127	.012	.027	.18	.135	.018	.136	.744
0.8	.524	.252	.113	.012	.028	.106	.135	0	.108	.714
0.9	.375	.214	.088	.012	.029	.075	.136	0	.091	.714
1.0	.208	.186	.074	.012	.03	.013	.094	0	.073	.684
1.1	.165	.14	.048	.012	.03	0	.094	0	.057	.65
1.2	.165	.097	.014	0	.03	0	.094	0	.038	.651
1.3	.165	.065	.005	0	.016	0	.049	0	.038	.488
1.4	.165	.045	.002	0	0	0	0	0	.02	.155
1.5	.009	.013	0	0	0	0	0	0	.02	.082

Table 7: Each column consists of the mean F-measure for a family for the specific threshold in the test set obtained from the multiple sequence alignment created using MAFFT. Similar trend of F-measure decline at threshold value of 0.4 is observed here.

thresh	tRNA	5s	SRP	tmRNA	RNaseP	16s	23s	GI	GII	telomerase
-0.2	.81	.568	.29	.243	.087	.541	.455	.074	.395	.812
-0.1	.81	.575	.282	.246	.087	.539	.455	.074	.395	.812
0.0	.817	.577	.359	.248	.09	.542	.46	.069	.4	.815
0.1	.867	.599	.365	.263	.098	.571	.453	.088	.486	.903
0.2	.886	.654	.372	.283	.098	.609	.5	.064	.638	.926
0.3	.885	.685	.383	.36	.091	.682	.554	.082	.7	.927
0.4	.898	.747	.395	.464	.099	.746	.626	.184	.785	.926
0.5	.947	.791	.421	.567	.115	.776	.817	.192	.855	.926
0.6	.97	.835	.444	.537	.109	.764	.837	.247	.897	.926
0.7	.982	.899	.551	.63	.113	.729	.886	.605	.951	.93
0.8	.984	.928	.555	.628	.167	.757	.885	0	.975	.929
0.9	.985	.941	.647	.628	.303	.764	.886	0	.978	.929
1.0	.988	.945	.656	.629	.819	.634	.885	0	.974	.93
1.1	.996	.955	.703	.627	.814	0	.885	0	.974	.927
1.2	.996	.966	.646	0	.0	0	.884	0	.962	.928
1.3	.996	.974	.4	0	.0	0	.872	0	.962	.92
1.4	.996	.974	.0	0	0	0	0	0	.972	.886
1.5	.008	.946	0	0	0	0	0	0	.973	.88

Table 8: Each column consists of the mean PPV for a family for the specific threshold in the test set obtained from the multiple sequence alignment created using MAFFT. Similar trend of PPV change in at threshold value of 0.4 is observed here.
